# Ancient duplication, coevolution, and selection at the MHC class IIA and IIB genes of birds

**DOI:** 10.3389/fimmu.2023.1250824

**Published:** 2023-10-27

**Authors:** Piotr Minias, Scott V. Edwards, Wiesław Babik

**Affiliations:** ^1^ University of Lodz, Faculty of Biology and Environmental Protection, Department of Biodiversity Studies and Bioeducation, Lodz, Poland; ^2^ Harvard University, Museum of Comparative Zoology, Cambridge, MA, United States; ^3^ Harvard University, Department of Organismic and Evolutionary Biology, Cambridge, MA, United States; ^4^ Jagiellonian University, Institute of Environmental Sciences, Faculty of Biology, Kraków, Poland

**Keywords:** birds, duplication, concerted evolution, major histocompatibility complex, MHC, orthology

## Abstract

**Introduction:**

The Major Histocompatibility Complex (MHC) of vertebrates is a dynamically evolving multigene family primarily responsible for recognizing non-self peptide antigens and triggering a pathogen-specific adaptive immune response. In birds, the MHC was previously thought to evolve via concerted evolution with high degree of gene homogenization and the rapid loss of orthology. However, the discovery of two ancient avian MHC-IIB gene lineages (DAB1 and DAB2) originating before the radiation of extant birds indicated that despite the action of concerted evolution, orthology may be detectable for long evolutionary periods.

**Methods:**

Here, we take advantage of rapidly accumulating digital genomic resources to search for the signal of an ancient duplication at the avian MHC-IIA genes, as well as to compare phylogenetic distribution and selection between MHC-IIA and IIB gene lineages.

**Results:**

The analysis of MHC sequences from over 230 species representing ca. 70 bird families revealed the presence of two ancient MHC-IIA gene lineages (DAA1 and DAA2) and showed that their phylogenetic distribution matches exactly the distribution of DAB1 and DAB2 lineages, suggesting tight coevolution. The early post-duplication divergence of DAA1 and DAA2 was driven by positive selection fixing radical amino acid differences within the membrane-proximal domain and, most probably, being functionally related to the interactions between α2 and β2 chains of the MHC-II heterodimer. We detected no evidence for an overall (gene-wide) relaxation or intensification of selection at either DAA1/DAB1 or DAA2/DAB2, but codon-specific differences in selection signature were found at the peptide-binding sites between the two gene lineages, perhaps implying specialization to different pathogen regimes.

**Discussion:**

Our results suggest that specific pairing of MHC-II α and β chains may have an adaptive significance, a conclusion that advances knowledge on the macroevolution of the avian MHC-II and opens exciting novel directions for future research.

## Introduction

The Major Histocompatibility Complex is a gene-dense region [or regions, as in teleost fishes (1)] in the genomes of all jawed vertebrates (2) and contains genes involved in both the immune response and in other functions (3). The most prominent among these genes are the eponymous Major Histocompatibility Complex genes – glycoproteins expressed on the cell surface that present peptides, derived from the degradation of self and foreign proteins, to T cells carrying variable receptors generated by somatic recombination (4). Recognition of non-self-peptide antigens triggers a pathogen-specific adaptive immune response (5). The two MHC classes likely share a common ancestry (6), but differ in both structure and role in the immune response. Although both MHC class I (MHC-I) and class II (MHC-II) are dimers, the structure of MHC-I and MHC-II dimers is profoundly different (5). In MHC-I, which mainly presents antigenic peptides derived from viruses and intracellular bacteria, the peptide binding cleft is formed by a single transmembrane chain, α, encoded by highly polymorphic gene(s) located in the MHC region, which associates with the non-polymorphic β_2_-microglobulin encoded by a gene located outside the MHC region. The MHC-II, which presents mainly antigenic peptides derived from extracellular pathogens, is a heterodimer consisting of two structurally similar transmembrane protein chains – α and β. The domains α1 and β1 of these chains together form the peptide binding cleft and both α (IIA) and β (IIB) genes are located within the MHC region. The central role the MHC-I and MHC-II play in adaptive immunity makes them a major target of the coevolutionary arms races with pathogens, which are considered to be the main drivers of the extraordinary MHC polymorphism and the rapid evolution of its genomic architecture (7).

MHC genes represent a paradigm of a dynamically evolving multigene family, with frequent duplications, pseudogenizations, and gene losses leading to a conspicuous variation in the number of loci and their genomic arrangement, both between and within species (8–10). Two major modes of evolution have been proposed for MHC genes: concerted evolution and birth-and-death evolution (11). Under concerted evolution, the divergence between paralogs is eroded by interlocus recombination and gene conversion, leading to the phylogenetic clustering of genes by species. In the birth-and-death model, duplicates evolve largely independently, but gene gains and losses are common, leading to complex relationships between genes and difficulties in establishing orthology at larger phylogenetic distances. It is becoming increasingly clear that these modes of MHC gene family evolution are not mutually exclusive and that both may operate within a single species (12), their relative importance possibly being time-dependent, with concerted evolution being more pronounced soon after duplication (13). Thus, we have a reasonable mechanistic understanding of the processes shaping variation in the genomic complexity of the MHC among taxa, reflecting net differences in the duplicate retention rate and gene homogenization rate via concerted evolution. It is less clear what are the ultimate biological drivers of the differences in the number of MHC genes and their divergence between taxa, but large-scale comparative studies (9, 10, 14, 15) and theoretical modelling (16) are beginning to provide interesting insights in this regard.

In mammals, MHC-I genes are thought to evolve and turnover rapidly, so that identification of orthology is generally not possible between eutherian orders (17, 18). In contrast, mammalian MHC-IIA and IIB genes, such as DP, DQ, or DR, often organized in the genome as tandemly duplicated IIA-IIB units, have evolved independently over long periods and can be identified as orthologs in most, though not all, eutherian taxa (19, 20). In birds, early studies suggested much more pronounced concerted evolution with the rapid loss of orthology, so that sequences from different MHC II genes formed species-specific clusters (21–23). The universality of this view was challenged by the discovery of two MHC-IIB genes (*DAB1* or *DAB2*) in owls, which retained orthology for long evolutionary periods (24, 25), and their origin was traced to the duplication predating the root of extant birds at > 100 million years ago (26). Both paralogs have been retained in many avian orders, though multiple cases of loss of either gene lineage have also been inferred (26). Despite their ancient origin, both genes have been experiencing a strong concerted evolution, which has led to a homogenization of sequences between the paralogs, except for the 5’ region of exon 3, which in most cases allows an unambiguous assignment of a given gene to either *DAB1* or *DAB2* lineage (25, 26). This part of the protein exhibits several radical amino acid substitutions changing basic to acidic residues, which were inferred to have been fixed by positive selection in the *DAB1* soon after the duplication that gave rise to both genes (25). The key region of the β chain that differentiates *DAB1* from *DAB2* is primarily involved in interaction with the α chain of the MHC-II molecule (25).

The pioneering work of Burri and colleagues (24–26) provided important insights into the molecular evolution and phylogenetic distribution of *DAB1* and *DAB2* genes in birds, but also raised further questions that we attempt to address in this paper. Since only a small part of IIB genes proved resistant to the homogenizing effects of gene conversion and since it encodes residues involved in the interaction between the α and β chains of the MHC-II molecule, testable predictions can be formulated on the evolution of IIA genes in birds. In particular, we expect the presence of two lineages of IIA orthologs, *DAA1* and *DAA2*, showing similar or identical phylogenetic distribution as *DAB1* and *DAB2*. We also expect *DAA1* and *DAA2* to show consistent divergence at the 5’ end of exon 3, caused by radical amino acid substitutions in *DAA1*. The expectations regarding the strength of concerted evolution and the resulting degree of sequence homogenization between *DAA1* and *DAA2* are less clear. The prediction of coevolving *DAB1*-*DAA1* and *DAB2*-*DAA2* pairs in birds is certainly plausible, but by no means trivial, as genomic arrangement and duplication patterns of IIA and IIB genes vary across vertebrate phylogeny. In birds alone, four genomic IIA and IIB arrangements have been described, each of which occurs in multiple phylogenetic lineages (12, 27). A simple avian MHC architecture is often characterized by a single tightly linked IIA-IIB pair or a single IIA gene located at a considerable genomic distance from IIB (27). However, an increasing complexity of the MHC region evolved either through the appearance of several tandemly duplicated IIA-IIB units, suggesting specific pairing of α and β chains and possibly their coevolution, or through the retention of a single IIA apparently serving multiple duplicated IIB genes. Passerine birds may represent an extreme case of the last scenario, as they typically have a single IIA gene, despite tens or more IIB genes (12, 27). The analysis of long-read genome assemblies revealed that, in fact, many non-passerine avian lineages also have a single IIA gene (27). However, there is a risk that IIA orthologs might have been collapsed in some assemblies, especially if their sequences were homogenized by concerted evolution (28), leading to a possible underestimation of the true number of IIA genes.

Another important question that has not yet been addressed is the mode and strength of selection acting on the peptide-binding domains (encoded by exon 2) of different evolutionary lineages of MHC-II genes (i.e. *DAA1* vs. *DAA2* and *DAB1* vs. *DAB2*). Although the hypervariable exons 2 of *DAB1* and *DAB2* have been largely homogenized by gene conversion (25, 26), this does not automatically imply the same selective pressure on both genes. Also, selection may either favour monomorphism or polymorphism of MHC-IIA genes, depending on the degree of their recombinational separation from MHC-IIB genes (29, 30). So far, the only comparative analysis of the second exon of avian IIA genes suggested predominantly purifying selection (27). Given the results of the recent synthesis suggesting substantial polymorphism and widespread adaptive evolution of IIA genes (31), a thorough investigation of adaptive evolution in the second exon of IIA genes is warranted, with particular emphasis on the possible differences among diverged IIA lineages.

In this study we integrate information from available genome assemblies and targeted resequencing projects to achieve the following specific goals: i) present an updated picture of the phylogenetic distribution of *DAB1* and *DAB2* across the tree for modern birds; ii) test the hypothesis that the pattern of loss of IIB genes is mirrored by the corresponding losses of IIA genes; iii) test whether a bout of positive selection mirroring that previously described for *DAB1* occurred in the *DAA1* lineage following the duplication; and iv) test for the differences in patterns of adaptive evolution between IIA and IIB genes and between paralogs of IIA and IIB.

## Methods

### Genomic resources

We primarily relied on publicly available genomic resources to retrieve MHC class IIA and IIB sequences across different clades of non-passerine birds (GenBank numbers and sequences provided in **Appendix 1**, **2**, respectively). Because the ancient MHC-IIB divergence was originally resolved in owls (24, 25), we generated a consensus MHC-IIB query from the orthologous *DAB1* and *DAB2* exon 3 sequences (282 bp) retrieved from a true owl species, the tawny owl *Strix aluco* (GenBank nos. KJ162540 and KJ162542, respectively). Because no owl MHC-IIA sequences were available in the Nucleotide Database of the National Center for Biotechnology Information (NCBI, Bethesda, MD, USA), we generated a consensus MHC-IIA query from the available MHC-IIA exon 3 sequences retrieved from a procellariform species, the Leach’s storm petrel *Hydrobates leucorhous* (GenBank nos. MN061391-MN061408). Both queries were BLASTed against 370 genomes of non-passerine birds available in the Genome NCBI Database (as accessed in November 2022), including a considerable number of long-read assemblies, which greatly improves the resolution of genomic features (32). Due to long divergence times between different non-passerine clades, we used the *blastn* algorithm, which was designed to search for short queries of moderate similarity in a cross-species framework. We only retained contigs containing complete or nearly complete (>90% of query coverage) exon 3 sequences. We also searched the Nucleotide NCBI Database for avian MHC-IIA and MHC-IIB exon 3 sequences obtained via targeted sequencing and these searches primarily focused on avian orders with low availability of genomic data. We extracted exon 3 sequences from all the contigs and aligned them by order in Geneious v.10.0.5 software (Biomatters Ltd., Auckland, New Zealand). Alignments from all palaeognath orders were merged into a single alignment due to low sample size within each order, while alignments from neognath orders were kept separate. All alignments were trimmed to full codons and we also removed the first (5’-end) and the last (3’-end) codons, because they were missing in some alignments, retaining a uniform 276 nt length of exon 3 across all orders. We removed highly similar (>98% of pairwise nucleotide similarity) sequences within each species (ca. 40% of all originally aligned sequences) and we only retained alignments with at least three MHC-IIA and MHC-IIB sequences originating from at least two species per order for the downstream analyses. Based on this selection criteria, we retained a single combined alignment for Palaeognathae (n = 4 orders) and 17 alignments for different orders of non-passerine Neognathae birds. Our final dataset comprised 184 MHC-IIA and 268 MHC-IIB exon 3 sequences originating from 235 species (156 and 208 species for MHC-IIA and IIB, respectively) and representing 71 avian families. The wider phylogenetic coverage of MHC-IIB than MHC-IIA sequences was primarily attributed to their better availability in the Nucleotide NCBI Database (MHC-IIA are largely understudied in birds), rather than to better retrieval rate from genomic resources.

### Phylogenetic clustering of membrane-proximal domains (exon 3)

To resolve clusters of orthologous MHC-IIA sequences within our alignments we used Bayesian tree-building algorithms, as implemented in MrBayes v.3.2.6 (33). Since orthology of MHC-IIB sequences was originally resolved in owls (24), we started phylogenetic analysis of MHC-IIA exon 3 sequences from this avian order. Prior to phylogenetic analysis, selection of an appropriate model of nucleotide substitution was performed in Mega v.6.0 software (34) using maximum likelihood and the lowest Bayesian Information Criteria (BIC). The Chinese giant salamander *Andrias davidianus* class IIA gene was used as outgroup (Genbank no. KF611869). Phylogenetic analysis was conducted using two independent chains, each consisting of one million iterations, burin-in length of 250 000 steps/iterations, and sampling every 500^th^ tree, yielding an expected sample size of 1500 retained trees per chain. The analyses were run using the Molecular Clock with Uniform Branch Lengths models and the default uninformative flat priors. We also tested models with Unconstrained and Exponential Branch Lengths across selected avian orders, but they provided qualitatively similar tree topologies (results not shown). The average effective sample size for model parameters was 1453 ± 17 [SE]. Convergence of independent chains was confirmed by the average standard deviation of split frequencies (ASDSF) approaching zero (35) and the potential scale reduction factors (PSRF) approaching one (36). Extended majority-rule consensus topologies were determined using Geneiuos v.10.0.5.

Since phylogenetic analysis of MHC-IIA sequences in owls provided support for the presence of two lineages of orthologous sequences (henceforth referred to as *DAA1* and *DAA2*; see results for details), we have used the same protocols as described above to resolve MHC-IIA orthology in all other avian orders. Blakiston’s fish owl *Bubo blakistoni* sequences (contig GenBank nos. BJCB01040766 and BJCB01033660, respectively; genome assembly GenBank no. GCA_004320225) were added to each alignment as reference *DAA1* and *DAA2* sequences. Since all amino acid differences between consensus owl *DAA1* and *DAA2* sequences concentrated in the upstream (5’-end) 90 nt region of exon 3, we expected this part of the sequence to yield the highest power to resolve orthology of MHC-IIA genes in other bird orders. In fact, the same pattern was described for MHC-IIB exon 3, where amino acid differences between *DAB1* and *DAB2* sequences also concentrated in the upstream region (24, 26). Taking all this into account, all phylogenetic analysis was based on this short upstream fragment (90 nt) of MHC-IIA exon 3. An assignment of MHC-IIA sequences to either *DAA1* or *DAA2* gene lineage was based on their monophyletic clustering with reference *DAA1* and *DAA2* sequences. Since nucleotide sequences provided poor cluster support (<0.50) in Psittaciformes, we used amino acid sequences to resolve orthology in this clade.

The evolutionary history of both orthologous gene lineages was reconstructed along the bird tree topology developed by Prum et al. (37) and visualized using the Interactive Tree Of Life (iTOL) v.5.0 webserver (38). We also tested whether phylogenetic resolution of *DAA1* and *DAA2* was retained across the remaining part of MHC class IIA exon 3. For this purpose, we conducted phylogenetic analysis of the central and downstream region (186 nt) of exon 3 in three selected avian orders with extensive coverage (at least ten available sequences per order), all showing evidence for the presence of *DAA1* and *DAA2* in the analysis of the upstream region of exon 3 (Charadriiformes, Procellariiformes, and Coraciiformes).

Although the history of the ancient duplication at the MHC-IIB has already been tracked throughout the evolution of birds (26), we aimed to assign all the retrieved MHC-IIB sequences to either *DAB1* or *DAB2*. Similarly to MHC-IIA, we focused on the upstream (5’-end) 90 nt region of exon 3 in the phylogenetic inference. We used Bayesian phylogenetic analysis algorithms, as described above. Tawny owl sequences (GenBank nos. KJ162540 and KJ162542) were added to each alignment as *DAB1*/*DAB2* reference and the Chinese giant salamander was used as outgroup (Genbank no. KF723002).

Our phylogenetic analyses were only used to identify the occurrence (presence or absence) of gene lineages at both MHC-IIA and MHC-IIB (*DAA1*/*DAB1* and *DAA2*/*DAB2*) and we did not aim to quantify gene copy number within each lineage. The occurrence of gene lineages was inferred on the level of bird orders (Neognathae) or across orders (Palaeognathae). We did not aim to characterize gene lineage occurrence at the species level, as technical artefacts (sequences missing in low-quality genome assemblies) could be easily confused with a true absence (loss) of either gene lineage.

### Retrieval of the peptide-binding domains (exon 2)

From the contigs containing either MHC-IIA or MHC-IIB exon 3 sequences, for which orthology was successfully resolved via phylogenetic inference, we also retrieved sequences of exon 2 encoding the peptide-binding α1 and β1 domains of the MHC-II molecule. In total, we retrieved 157 MHC-IIA and 215 MHC-IIB full length (255 and 270 nt, respectively) sequences of exon 2 across the entire dataset. All retrieved sequences encoded putatively functional proteins (lacked stop codons or frame-shift mutations).

### Inferring positive selection

All retrieved MHC-IIA and IIB exon 2 and 3 sequences were used to quantify and compare sequence diversity and selection between membrane-proximal (α2 and β2) and peptide-binding (α1 and β1) domains of *DAA1*/*DAA2* and *DAB1*/*DAB2*. For this purpose, we prepared combined alignments across all avian orders for each exon (separately for each gene and gene lineage) and we first used DnaSP v6.10.03 (39) to quantify sequence diversity (as measured with nucleotide diversity and the total number of mutations). Second, we removed putative recombinant sequences from the alignments, since under high recombination rate no unique tree topology can describe evolutionary history of entire sequences (40), which may lead to biases in selection estimates. We used RDP v.4.80 software (41) to assess recombination signal using seven different algorithms (all available except for the most computationally demanding LARD approach). All analyses were run using default settings and recombination events were identified if supported by at least three different approaches. An inference of recombination signal at each exon within each gene lineage was conducted separately for each avian order (except of palaeognath birds which were analysed jointly). In total, 11.3% sequences showed recombination signal (1.4-18.5% sequences per exon per gene lineage) and they were all removed from the final alignments prior to the analyses of selection patterns. However, because inferring recombination between species (within orders) may produce false positives (e.g. homoplasy could yield a similar signal as recombination), we also re-ran all selection inferences using the entire dataset (no putative recombinant sequences removed). The estimates of selection (*dN* and *dS*, see below for details) for each exon from each gene lineage were highly consistent between the two datasets (average Pearson correlation coefficient r = 0.94 ± 0.02 [SE]) and, thus, we concluded that any possible removal of false positive recombinant sequences from the alignments was unlikely to introduce any major bias in the results. Our final alignments (after removal of recombinant sequences) mostly consisted of single sequences per species and only ca. 5% sequences were identified as multiple replicates within species (max. 3 sequences per species). To test if the presence of multiple sequences per species biased our selection inferences, we randomly retained a single sequence per species in each alignment and re-ran the analyses. The *dN* and *dS* estimates were highly consistent between the two approaches (average r = 0.989 ± 0.003 [SE]), thus providing support for no biases.

The patterns of selection were quantified using the relative rate of nonsynonymous nucleotide substitutions at non-silent sites (*dN*), which are a target of selection, to synonymous substitutions at silent sites (*dS*), which are presumably neutral (42). In general, sustained positive (diversifying) selection promotes changes in the protein sequence and, thus, nonsynonymous substitutions accumulate faster than synonymous substitutions (*dN*/*dS* > 1). In contrast, negative (purifying) selection suppresses protein changes, so nonsynonymous substitutions are removed and accumulate at a slower rate than synonymous substitutions (*dN*/*dS* < 1). To infer codon-specific signature of pervasive, i.e. constant across the entire tree (affecting all avian lineages) positive and negative selection we used Fast Unconstrained Bayesian AppRoximation (FUBAR) (43), as implemented in the Datamonkey v.2.0 webserver (44). This approach is based on a Markov Chain Monte Carlo (MCMC) routine, which is relatively robust against model misspecifications and leaves the distribution of selection parameters essentially unconstrained (43). Mixed Effect Model of Evolution (MEME) approach (45) was used to infer signature of episodic positive selection, which is apparent at a proportion of tree branches (i.e. affecting only some avian lineages). We also used Contrast Fixed Effects Likelihood (contrast-FEL) method (46) to identify codons showing differences in selection signature (*dN*/*dS* ratio) between the two gene lineages (across both domains of either MHC-IIA or IIB gene). All these analyses were run using default settings and input trees inferred directly from the alignments. Finally, we used branch-specific FEL approach (47) to test if positive selection was associated with the early post-duplication divergence of *DAA1* and *DAA2* exon 3, as previously shown for *DAB1* and *DAB2* (25). For this purpose, we searched for the signature of positive selection at the branch separating *DAA1* from *DAA2* lineages. Although this analysis was conducted for the entire exon 3, the input tree was based on the upstream 90 nt region, which provided a good resolution of *DAA1* and *DAA2* clusters. Codons (sites) under selection were identified based on posterior probabilities >0.95 (FUBAR) and P values <0.05 (MEME, FEL, and contrast-FEL).

The *dN*/*dS* ratios (as estimated with FUBAR) were compared between genes (MHC-IIA and IIB), gene lineages (*DAA1*/*DAA2* and *DAB1*/*DAB2*), exons within each gene lineage (exon 2 and 3), and regions within each exon. We distinguished two regions within exon 3: *i*) the upstream region (90 nt), which was used in phylogenetic analysis to resolve orthology and is primarily involved in interactions between the two membrane-proximal (α2 and β2) domains of the MHC-II molecule, and *ii*) the remaining central and downstream region (186 nt), which is more involved in interactions with the peptide-binding domains, but also forms a conserved hydrophobic pocket interacting with CD4 T cell coreceptor (β2 domain only). Positions of codons involved in different types of interactions (between membrane-proximal domains, with peptide-binding domains, or with CD4 coreceptor) were inferred based on the crystallographic analyses of MHC-II molecules in humans and chickens *Gallus gallus* (48–50). Within exon 2 we also distinguished two classes of codons: *i*) the peptide-binding sites (PBS), which are directly involved in interactions with antigens, and *ii*) the non-peptide binding sites (non-PBS) comprising all the remaining codons. Positions of putative PBS codons were inferred based on the crystallographic analyses of MHC-II molecules in humans (51).

### Statistical analyses

We used general linear models (GLM) to test for differences in selection signature (*dN*/*dS* ratio) between genes, gene lineages, exons, and exonic regions. Codon-specific estimates of *dN* and *dS* we log-transformed to improve normality (final skewness <1) and log *dN*/*dS* ratio was entered as a response variable in the models. First, separate GLMs were run for both exons (2 and 3) and included gene, gene lineage, and exonic region as fixed factors. We also included all possible two-way interactions between fixed factors, but they were removed from the final models if non-significant. Second, to test for the differences in selection between both exons we ran two separate GLMs for MHC-IIA and IIB genes, including exon identity as a fixed factor. All GLMs were run in Statistica v.13.0 (Tibco Software Inc., Palo Alto, CA, USA). We also used G tests to detect differences in the proportion of positively and negatively selected sites between gene lineages of MHC-IIA and IIB. Finally, we used the RELAX approach (52) available at the Datamonkey webserver to test for the relaxation or intensification of selection between gene lineages. Although this method does not explicitly test for positive selection, it uses likelihood ratio tests (LRTs) to identify shifts in the stringency of selection between two subsets of branches (herein corresponding to both gene lineages).

## Results

### Phylogenetic analysis

**Figure 1:**
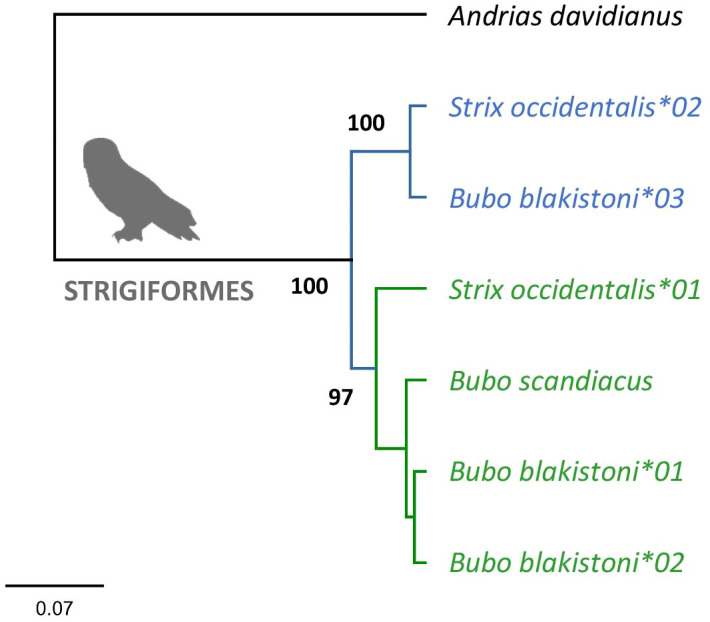
Consensus Bayesian topology of MHC class IIA sequences in owls Strigiformes. Inferred *DAA1* and *DAA2* clusters are marked in green and blue, respectively. Phylogenetic relationships were assessed for the full length (282 bp) nucleotide sequences of MHC-IIA exon 3. Bayesian posterior probabilities were provided for major clusters. *Andrias davidianus* was used as outgroup (GenBank no. KF611869).

Phylogenetic analysis of MHC class IIA exon 3 sequences in Strigiformes provided evidence for two distinct clusters of sequences yielding very high consensus support (**Figure 1**). Since the sequences clustered by their apparent orthology rather than by species and since more than one sequence retrieved per genome indicated gene duplication, we considered these clusters to represent two MHC class IIA gene lineages (*DAA1* and *DAA2*).

**Figure 2:**
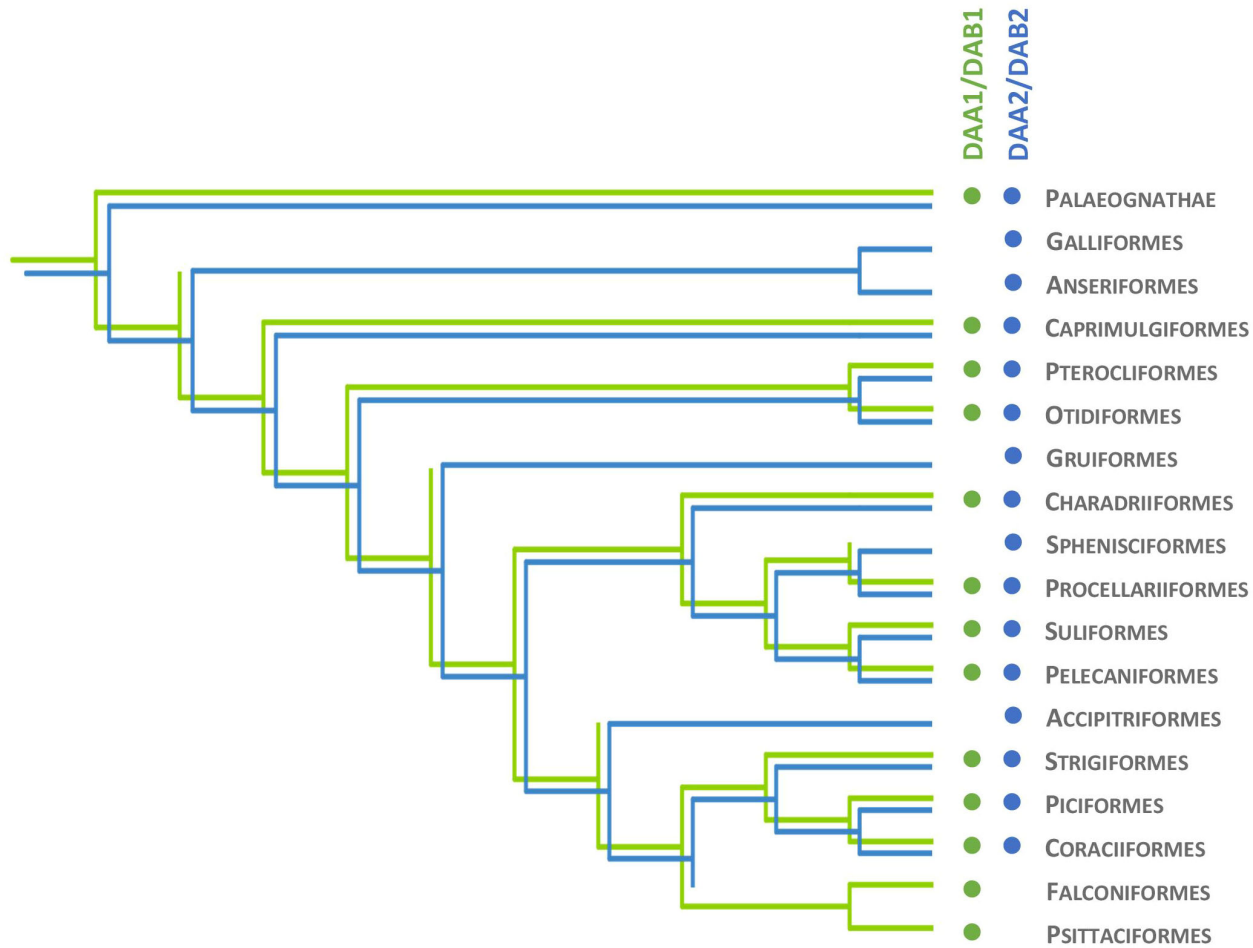
Evolutionary history of MHC class IIA (*DAA1* and *DAA2*) and MHC class IIB (*DAB1* and *DAB2*) genes in non-passerine birds. Presence of *DAA1/DAB1* and *DAA2/DAB2* gene lineage is marked with green and blue, respectively.

The clustering of MHC-IIA exon 3 sequences (upstream region) in the basal avian clade Palaeognathae revealed the presence of both *DAA1* and *DAA2* (**Appendix 3**). The *DAA1* and *DAA2* clusters were monophyletic with moderately high to very high posterior probability (**Appendix 2**, **3**). Assignment of particular sequences to each cluster revealed the presence of both *DAA1* and *DAA2* in Tinamiformes, whereas the other orders showed the presence of either *DAA1* (Casuariiformes) or *DAA2* (Rheiformes and Apterygiformes). Phylogenetic analysis of MHC-IIA exon 3 sequences in non-passerine neognath birds revealed the presence of both *DAA1* and *DAA2* clusters in 10 out of 17 orders (**Figure 2**). In five orders we recorded only the *DAA2* gene cluster and these included both early-branching (Galliformes and Anseriformes) and more derived non-passerine avian clades (Gruiformes, Sphenisciformes, and Accipitriformes) (**Figure 2**). Whereas the absence of *DAA1* sequences in Galliformes and Anseriformes suggests that the loss of *DAA1* occurred prior to the split of Galloanserae into these two major lineages, the other clades were scattered across the avian phylogeny, suggesting multiple independent *DAA1* losses (**Figure 2**). At the same time, only the *DAA1* cluster was detected in the two most derived non-passerine clades (Falconiformes and Psittaciformes) (**Figure 2**), indicating a single major transition from *DAA1*/*DAA2* to *DAA1* in the evolution of avian MHC-IIA. Bayesian posterior probabilities for monophyletic *DAA1* or *DAA2* clusters were in most cases high or very high (>0.90; **Appendix 3**).

Whereas the upstream region of MHC class IIA exon 3 provided a good phylogenetic resolution of *DAA1* and *DAA2* genes across diverse avian orders, this signal was not retained across the remaining part of exon 3 (central and downstream region). Phylogenetic analysis of these sequences in three selected bird orders (Charadriiformes, Procellariiformes, and Coraciiformes) revealed mixed clustering of *DAA1* and *DAA2* and relatively high consensus support for many mixed *DAA1*/*DAA2* clusters, allowing no reliable resolution of orthology (**Appendix 4**), and suggesting concerted evolution across the central and downstream regions of exon 3.

**Figure 3:**
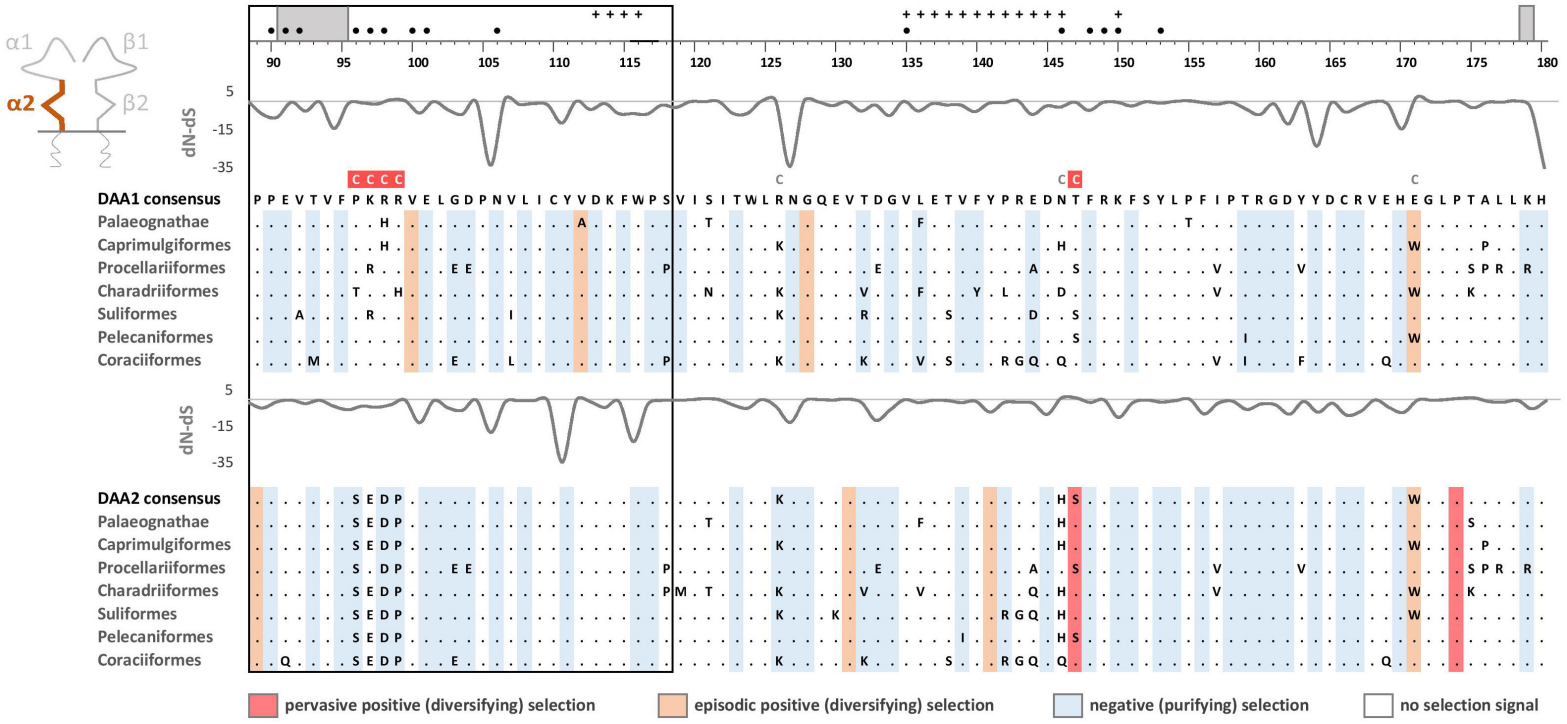
Alignments of amino acid sequences of MHC class IIA (*DAA1* and *DAA2*) exon 3 (α2 domain) in non-passerine birds (consensus sequences shown for Palaeognathae and six selected Neognathae bird orders). Dots indicate amino acids identical with the reference consensus *DAA1* sequence (as inferred using all available sequences). Positively selected residues are marked with dark red (pervasive selection) or light red (episodic selection), while negatively selected residues are marked with blue (as inferred for non-recombinant sequences using FUBAR and MEME approaches). Variation in selection parameter (*dN*–*dS*) is shown above each alignment. Amino acid differences between *DAA1* and *DAA2* consensus sequences were marked with C above the upper (*DAA1*) alignment (marked on red background if showing signature of early post-duplication positive selection). Residues putatively involved in interactions with β2 domain (filled black bullets), peptide-binding β1 domain (crosses), and CD4 coreceptor (grey bars) were marked at the top of the figure [all following Murthy and Stern (41), Wang et al. (42), Zhang et al. (43)]. Upstream region used for phylogenetic inference was framed with black line.

**Figure 4:**
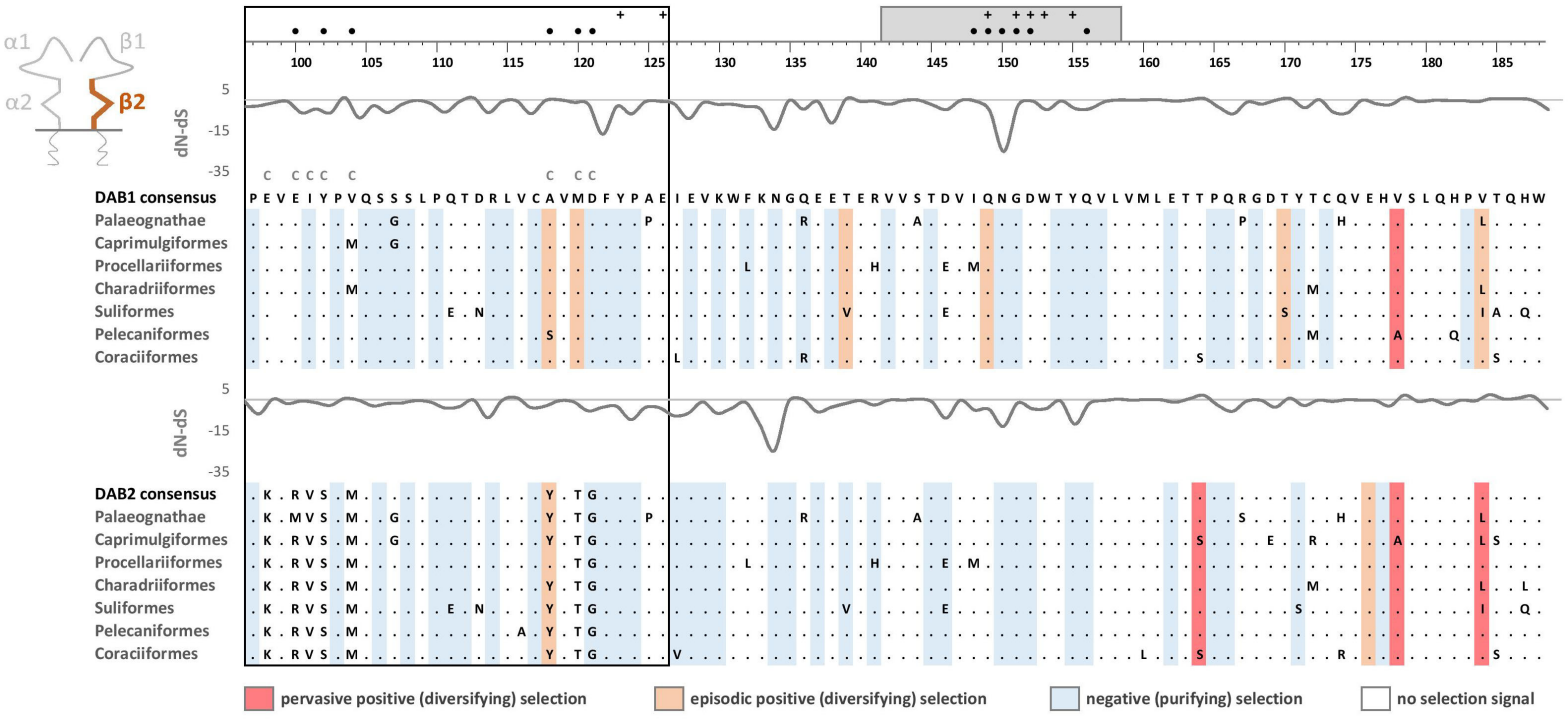
Alignments of amino acid sequences of MHC class IIB (*DAB1* and *DAB2*) exon 3 (β2 domain) in non-passerine birds (consensus sequences shown for Palaeognathae and six selected Neognathae bird orders). Dots indicate amino acids identical with the reference consensus *DAB1* sequence (as inferred using all available sequences). Positively selected residues are marked with dark red (pervasive selection) or light red (episodic selection), while negatively selected residues are marked with blue (as inferred for non-recombinant sequences using FUBAR and MEME approaches). Variation in selection parameter (*dN*–*dS*) is shown above each alignment. Amino acid differences between *DAB1* and *DAB2* consensus sequences were marked with C above the upper (*DAB1*) alignment. Residues putatively involved in interactions with α2 domain (filled black bullets), peptide-binding α1 domain (crosses), and CD4 coreceptor (grey bar) were marked at the top of the figure [all following Murthy and Stern (41), Wang et al. (42), Zhang et al. (43)]. Upstream region used for phylogenetic inference was framed with black line.

Phylogenetic analysis of MHC-IIB exon 3 sequences revealed identical patterns of duplication as found for MHC-IIA (**Appendix 3**), suggesting coevolution of these genes throughout the evolution of birds (**Figure 2**). Phylogenetic analysis of MHC-IIB also generally agreed with the results previously presented by Goebel et al. (26), although our analyses lent support for the presence of both *DAB1* and *DAB2* in some clades, which were previously reported to lack either of gene lineages (Pterocliformes, Otidiformes, Coraciiformes). It was noteworthy that positions of codons most divergent between the two gene lineages (showing amino acid differences between consensus sequences of orthologous gene lineages inferred across all avian orders) clearly overlapped with positions of sites responsible for interactions between the membrane-proximal domains of MHC-IIα and β subunits (**Figure 3**). Six out of eight divergent codons of the α2 domain overlapped or were adjacent to sites putatively involved in interactions with β2 domain, and five of them showed signal of positive selection along the branch separating *DAA1* and *DAA2*, indicating that the early post-duplication divergence of the α2 domain was likely driven by positive selection. Consistent with this pattern, seven out of eight divergent codons at β2 domain overlapped with sites putatively involved in interactions with α2 domain (**Figure 4**). There was a minimal overlap of the divergent codons with sites responsible for other types of interactions (with the peptide-binding domain or CD4 coreceptor) (**Figures 3**, **4**), indicating that functional divergence of MHC-IIA and IIB gene lineages is primarily manifested in the interactions between α2 and β2 domains.

### Selection at the membrane-proximal α2 and β2 domains

**Figure 5:**
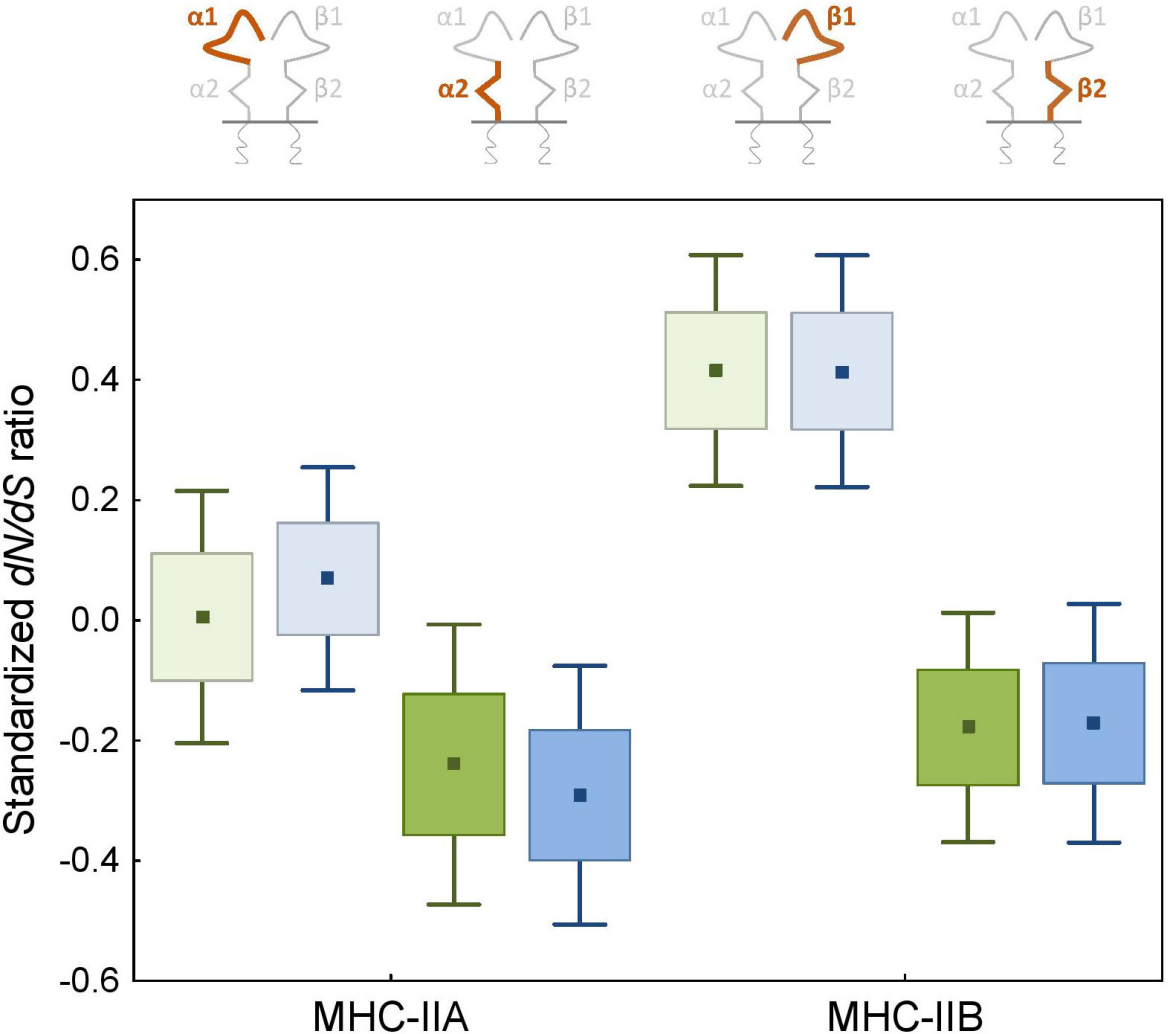
Signature of selection (standardized *dN*/*dS* ratio) at the peptide-binding α1 and β1 (light colours) and membrane-proximal α2 and β2 (bright colours) domains of the MHC class II genes in non-passerine birds. *DAA1*/*DAB1* and *DAA2*/*DAB2* gene lineages are marked with green and blue, respectively.

Pervasive negative (purifying) selection was identified as the key evolutionary force acting at α2 and β2 domains encoded by MHC-II exon 3. Between 37% and 44% sites of the membrane-proximal domains showed signature of negative selection (**Table 1**; **Figures 3**, **4**). In contrast, no more than three sites were found to be under pervasive positive (diversifying) selection, while an additional 2-5 sites were under episodic positive selection, meaning that on average only 5.7% sites at these domains showed any signature of positive selection (**Table 1**; **Figures 3**, **4**). The signature of selection was unevenly distributed along exon 3 sequence, as *dN*/*dS* ratio was significantly lower in the upstream region used for phylogenetic analysis (stronger negative selection) compared with the remaining central/downstream regions (F_1,364_ = 4.17, P = 0.042). Stronger negative selection in the upstream region was apparent across both pairs of MHC class IIA and IIB gene lineages, as indicated by non-significant region-gene (F_1,361_ = 0.79, P = 0.37) and region-lineage (F_1,361_ = 0.01, P = 0.91) interactions. We found no significant differences in the mean strength of selection (*dN*/*dS* ratio) at the membrane-proximal domains between MHC class IIA and IIB genes (F_1,364_ = 0.74, P = 0.39) or between gene lineages (F_1,364_ = 0.04, P = 0.83; gene-lineage interaction: F_1,361_ = 0.07, P = 0.78) (**Figure 5**). Also, the proportion of positively and negatively selected sites did not differ between gene lineages (*DAA1* vs. *DAA2*: G = 0.08, P = 0.78; *DAB1* vs. *DAB2*: G = 0.02, P = 0.89) and there was no evidence for a relaxation or intensification of selection at *DAA1* vs. *DAA2* (LR = 0.00, P = 1.00) and *DAB1* vs. *DAB2* (LR = 0.88, P = 0.35). Despite similar overall strength of selection, we identified codon-specific differences in *dN*/*dS* ratio at the membrane-proximal domains of both gene lineages. These differences were more apparent at the DAA genes, where we identified 12 codons showing evidence of different selection signature between *DAA1* and *DAA2*. In contrast, we identified only four codons showing different selection signature between *DAB1* and *DAB2* membrane proximal domains. Most of these codons (87.5%) showed higher *dN*/*dS* ratio at *DAA1*/*DAB1* than *DAA2*/*DAB2*.

**Table 1:** Sequence diversity and selection at the MHC class IIA (*DAA1* and *DAA2*) and MHC class IIB (*DAB1* and *DAB2*) exon 2 and 3 in non-passerine birds.

Gene	Region	Gene lineage	No. seq	No. species (families)	π	No. mutations	Number of codons under selection			*dN*/*dS*	
							Pervasive positive	Episodic positive	Negative	All codons	PBS codons
MHC-IIA	Exon 2 (α1)	*DAA1*	63 (53)	62 (30)	0.244	403	2	8	25	0.23	0.22
		*DAA2*	94 (84)	91 (41)	0.205	423	2	11	29	0.33	0.41
	Exon 3 (α2)	*DAA1*	72 (71)	68 (32)	0.176	292	0	4	36	0.14	–
		*DAA2*	112 (108)	103 (47)	0.152	385	2	4	41	0.16	–
MHC-IIB	Exon 2 (β1)	*DAB1*	97 (91)	91 (32)	0.236	457	8	9	27	0.76	1.39
		*DAB2*	118 (107)	105 (36)	0.248	514	12	13	25	0.76	1.21
	Exon 3 (β2)	*DAB1*	124 (101)	112 (38)	0.181	150	1	5	36	0.19	–
		*DAB2*	144 (117)	128 (45)	0.130	128	3	2	34	0.22	–

Sequence diversity was measured as nucleotide diversity (π) and the total number of mutations. The total number of sequences and the number of non-recombinant sequences (in parentheses) is provided. The number of codons under pervasive positive/negative and episodic positive selection were inferred using FUBAR and MEME approaches, respectively, for non-recombinant sequences only. The nucleotide substitution rate (dN/dS) was inferred across all codons (exon 2 and 3) and across the peptide-binding site (PBS) codons (exon 2).

### Selection at the peptide-binding α1 and β1 domains

**Figure 6:**
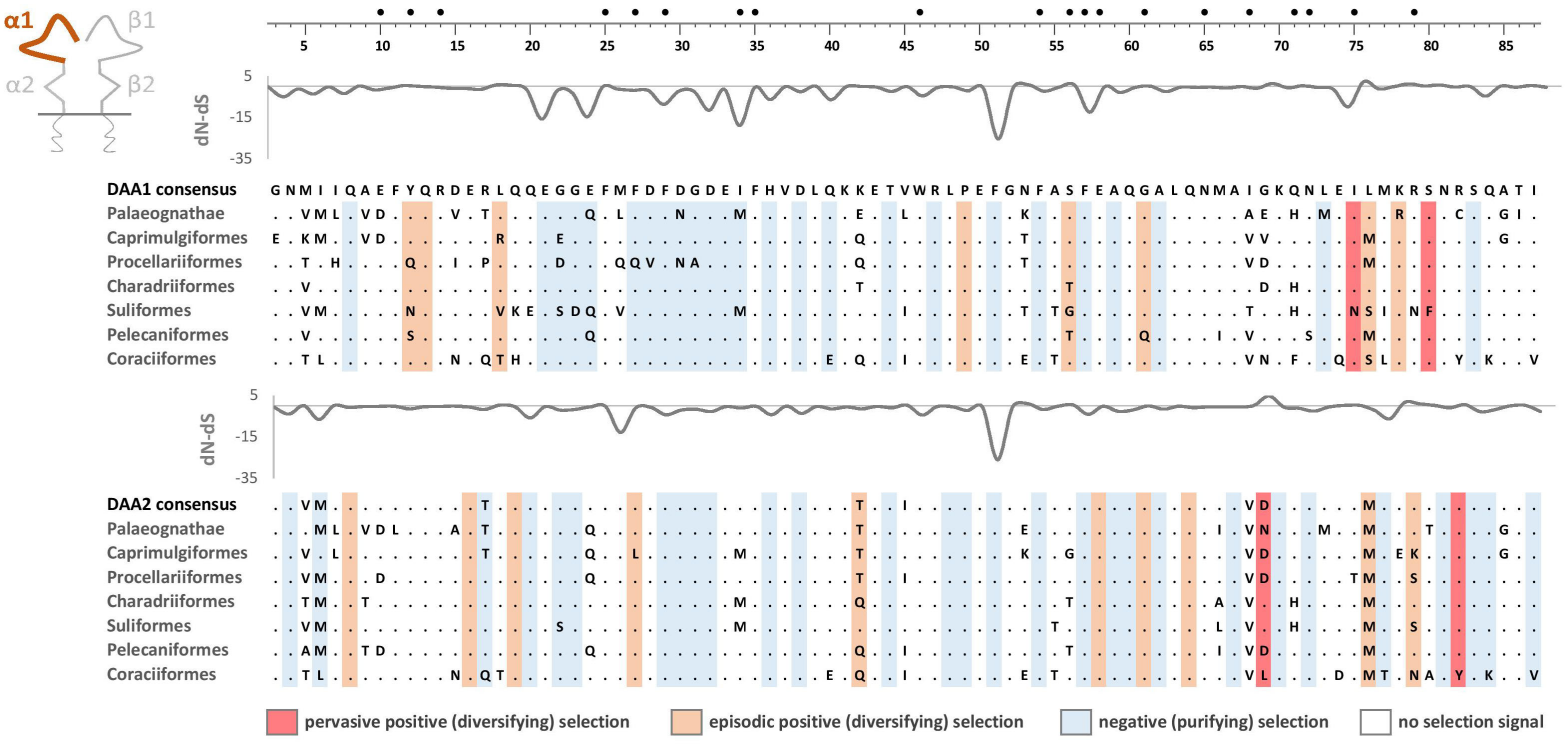
Alignments of amino acid sequences of MHC class IIA (*DAA1* and *DAA2*) exon 2 (α1 domain) in non-passerine birds (consensus sequences shown for Palaeognathae and six selected Neognathae bird orders). Dots indicate amino acids identical with the reference consensus *DAA1* sequence (as inferred using all available sequences). Positively selected residues are marked with dark red (pervasive selection) or light red (episodic selection), while negatively selected residues are marked with blue (as inferred for non-recombinant sequences using FUBAR and MEME approaches). Variation in selection parameter (*dN–dS*) is shown above each alignment. Putative peptide-binding residues were marked with filled black bullets at the top of the figure.

**Figure 7:**
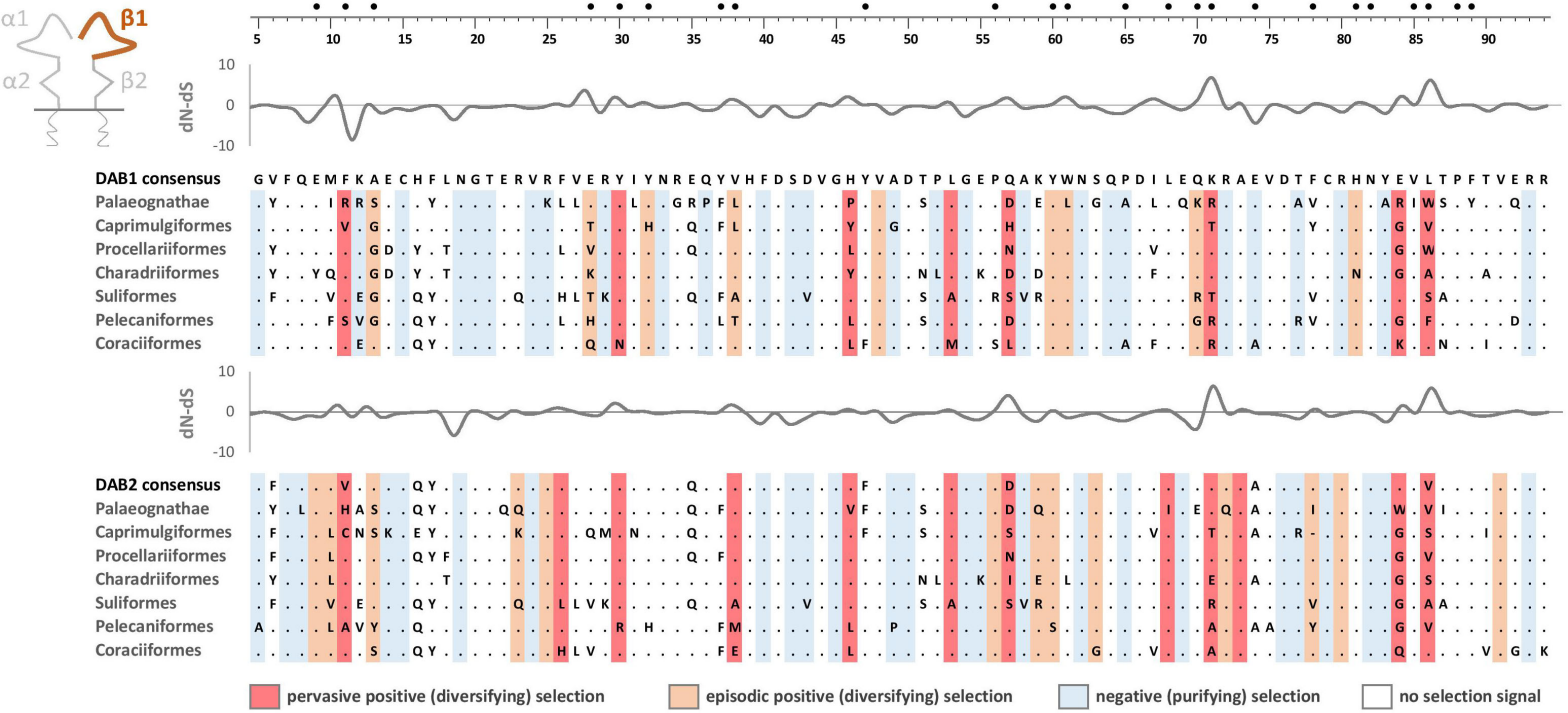
Alignments of amino acid sequences of MHC class IIB (*DAA1* and *DAA2*) exon 2 (β1 domain) in non-passerine birds (consensus sequences shown for Palaeognathae and six selected Neognathae bird orders). Dots indicate amino acids identical with the reference consensus *DAA1* sequence (as inferred using all available sequences). Positively selected residues are marked with dark red (pervasive selection) or light red (episodic selection), while negatively selected residues are marked with blue (as inferred for non-recombinant sequences using FUBAR and MEME approaches). Variation in selection parameter (*dN–dS*) is shown above each alignment. Putative peptide-b inding residues were marked with filled black bullets at the top of the figure.

As expected, the peptide-binding domains (encoded by exon 2) showed significantly stronger signature of positive selection than membrane-proximal domains, both at MHC-IIA (F_1,352_ = 8.01, P = 0.005) and MHC-IIB (F_1,362_ = 36.79, P < 0.001) genes (**Figure 5**). Nucleotide diversity was on average 45% higher at the peptide-binding than membrane-proximal domains (**Table 1**). Significantly stronger positive selection (higher *dN*/*dS*) was detected at β1 than α1 domain (F_1,347_ = 14.83, P < 0.001) (**Figure 5**). There were only two sites under pervasive positive selection at the α1 domains of *DAA1* and *DAA2*, although additional 8 (*DAA1*) and 11 (*DAA2*) codons showed signature of episodic positive selection (**Table 1**; **Figures 6**, **7**). In contrast, 17 (*DAB1*) and 23 (*DAB2*) codons were found to be under either pervasive or episodic selection at β1 domain (**Figures 6**, **7**). The number of negatively selected sites was similar between α1 and β1 domains (range: 25-29 at each gene lineage) (**Table 1**; **Figures 6**, **7**). Most of the positively selected sites (PSS) either overlapped with the putative PBS or were located at positions neighbouring to the PBS (a total of 65% and 78% PSS at α1 and β1 domain, respectively) (**Figures 6**, **7**). There were no differences in the mean strength of selection (*dN*/*dS* ratio) at the peptide-binding domains between gene lineages (F_1,347_ = 0.10, P = 0.76; gene-lineage interaction: F_1,346_ = 0.11, P = 0.74) (**Figure 5**) and the proportion of positively and negatively selected sites was also similar between lineage (*DAA1* vs. *DAA2*: G = 0.03, P = 0.87; *DAB1* vs. *DAB2*: G = 0.61, P = 0.43). We found no support for either relaxation or intensification of selection at the peptide-binding domains of *DAA1* versus *DAA2* (LR = 2.74, P = 0.10) and *DAB1* vs. *DAB2* (LR = 2.41, P = 0.12). However, similarly to the membrane-proximal domains, we found codon-specific differences in *dN*/*dS* ratio at the peptide-binding domains of both gene lineages. Specifically, contrasting selection signature was found at 10 codons for *DAA1*/*DAA2* and 12 codons for *DAB1*/*DAB2* gene lineages. The direction of these differences was highly variable between the codons, showing increased *dN*/*dS* at either *DAA1* (4 sites) or *DAA2* (6 sites) and either *DAB1* (4 sites) or *DAB2* (8 sites). Interestingly, 77.3% of codons with contrasting selection signature were located at positions directly overlapping or neighbouring with the putative PBS.

## Discussion

Our extensive analyses of available genomic data provided strong support for an ancient duplication of avian MHC-IIA that produced two gene lineages and suggested coevolution of MHC-IIA with MHC-IIB genes. We showed that phylogenetic distribution of MHC-IIA gene lineages (*DAA1* and *DAA2*) perfectly matches the distribution of the two MHC-IIB gene lineages (*DAB1* and *DAB2*). Although both gene lineages were retained in most non-passerine avian orders, we also recorded several independent losses of *DAA1*/*DAB1* lineages and a single loss of *DAA2*/*DAB2*. Second, we showed that the early post-duplication divergence of *DAA1* and *DAA2* was driven by positive selection fixing radical amino acid differences within the membrane-proximal domain of these genes (similarly to *DAB1* and *DAB2*). The functional significance of this divergence appears directly related to the interactions between α and β membrane-proximal domains, rather than to the interactions with peptide-binding domains or CD4 coreceptors. Although we detected no evidence for an overall relaxation or intensification of selection at either *DAA1*/*DAB1* or *DAA2*/*DAB2*, indicating that both gene lineages retained their functionality, we also recorded some codon-specific differences in selection signature at the peptide-binding sites between gene lineages (both at the MHC-IIA and IIB). This suggests that both MHC-II gene lineages may be subject to varying pathogen-driven selection regimes, possibly allowing to fine-tune their antigen binding properties.

The distribution of MHC-IIB gene lineages has already been characterized across the avian tree of life, but these analyses were primarily based on short sequences derived from targeted MHC genotyping, also being complemented with some early available (mostly short-read) genome assemblies (26). Both *DAB1* and *DAB2* gene lineages were originally detected in several avian orders (12 out of 34), but this scenario was actually suggested to be less prevalent than the occurrence of a single gene lineage recorded per order (*DAB1* and *DAB2* recorded as a single gene lineage in 9 and 12 orders, respectively). Based on these observations, Goebel et al. (26) concluded that this phylogenetic distribution may hint towards multiple independent losses of either of MHC-IIB lineages during the radiation of extant birds, but the presence of both MHC-IIB lineages in some orders may have also been masked by concerted evolution, which has often been invoked to explain the evolution of the avian MHC (22, 23, 53). The latter scenario was primarily supported by the observation of haplotypes displaying various degrees of intermingling between *DAB1* and *DAB2* lineages, suggesting concerted evolution through gene conversion events involving short sequence tracts (26). Here, we took advantage of the broad current availability of long-read genome assemblies, which enhanced resolution of *DAB1* and *DAB2* phylogenetic distribution in birds. In fact, we found both *DAB1* and *DAB2* in some avian orders that were previously reported to lack either of the gene lineages (Pterocliformes, Otidiformes, Coraciiformes). By focusing on non-passerine orders with adequate data (e.g., by excluding orders with single-species data) we concluded that the retention of both gene lineages was a dominant evolutionary scenario in birds and gene lineage losses (or gene lineage homogenization via concerted evolution) seems to be less prevalent than previously thought. At the same time, it needs to be explicitly acknowledged that gene lineage orthology was exclusively retained within the short upstream region of MHC-II exon 3, while the remaining regions of this exon were likely homogenized between the two gene lineages by concerted evolution. Similarly, concerted evolution is still expected to be a dominant force restraining divergence of duplicated genes within each gene lineage (either *DAA1*/*DAB1* or *DAA2*/*DAB2*). This mechanism could be especially important in passerine birds, which apparently retained only a single MHC-II gene lineage (25), but at the same time experienced extraordinary within-lineage duplication rates, producing tens of gene copies in some taxa (9, 50). In fact, the scenario of concerted evolution shaping evolutionary dynamics of the MHC-II was originally proposed for the passerines (21).

Although our analyses provided an updated picture of *DAB1*/*DAB2* distribution across the avian phylogeny, here we primarily focused on the evolution of MHC-IIA genes. Most importantly, we provided the first evidence for an ancient duplication of MHC-IIA genes in birds and showed that evolutionary trajectories and phylogenetic distribution of the two MHC-IIA gene lineages (*DAA1* and *DAA2*) perfectly matched the patterns described for *DAB1* and *DAB2*, suggesting a tight coevolution between these *DAA* and *DAB* gene lineages. Because the early post-duplication divergence at both MHC-IIA and MHC-IIB was driven by strong positive selection fixing radical amino acid differences at the sites responsible for the interactions between membrane-proximal α2 and β2 domains (demonstrated earlier by Burri et al. (25) for MHC-IIB), we conclude that this coevolution allows a specific pairing of α and β chains into two different types of MHC-II heterodimers. An astonishing conservation of this pattern across the evolutionary history of birds suggests that gene lineage divergence may have borne some important consequences for the structure and conformation of MHC-II molecule, although we acknowledge that this needs to be confirmed with modelling of protein structure. This corresponding phylogenetic distribution of MHC-IIA and IIB gene lineages and their tight coevolution also seem to lend support for gene loss scenario in avian lineages where only a single gene lineage was detected. As argued by Goebel et al. (26), strong intermingling between both gene lineages and homogenization of the entire membrane-proximal domains via gene conversion would imply that a functional divergence of the two lineages is unlikely. Here, we showed that this homogenization was effectively restrained in regions responsible for interactions between the membrane-proximal domains of MHC-IIA and IIB molecules, suggesting functional divergence in terms of MHC-II heterodimer assembly.

The gene loss scenario is further supported by the recent comparative analysis of genomic architecture of the avian MHC region (27). The inspection of high-quality genome assemblies obtained using long-read technologies revealed a consistent presence of a single MHC-IIA gene in nearly all avian orders (except Accipitriformes), where we detected the presence of only one gene lineage (either *DAA1*/*DAB1* or *DAA2*/*DAB2*), clearly supporting a loss of either MHC-IIA gene lineage (rather than the maintenance of two homogenized gene lineages). This could not have been observed at the level of MHC-IIB genes, which show much higher duplication rate compared to MHC-IIA, producing a pattern where a single MHC-IIA gene is linked with a tandem of duplicated MHC-IIB loci (3, 12). Although it has already been well-established that a single α chain can work effectively with different β chains (30), our analyses suggest that effective combinations likely occur only between specific MHC-IIA and IIB gene lineages, a hypothesis that could be tested experimentally. Whether duplication rates within *DAA1*/*DAB1* and *DAA2*/*DAB2* lineages are similar, or whether they have been intensified/relaxed in either lineage, still needs to be examined. It also remains an open question whether highly intermingled *DAB1*-*DAB2* haplotypes [as described by Goebel et al. (26)] retain their functionality, and if so, whether they can effectively pair with either *DAA1* or *DAA2* sequences, or whether they originate from loci that lost functionality through the birth-and-death evolutionary processes (11). Experimental evidence from model organisms including humans shows that mismatched (e.g. mixed-isotype) combinations of α- and β-chains may alter functionality of MHC class II molecules, but may also generate non-functional heterodimers that are either not expressed on cell surface or have impaired antigen binding functions due to misfolding (54–56).

The analysis of nucleotide substitution rates at the peptide-binding domains revealed no evidence for an overall intensification or relaxation of selection at either *DAA1*/*DAB1* or *DAA2*/*DAB2* gene lineage, indicating that both gene lineages likely retained their functionality in terms of pathogen recognition. However, we have found conspicuous codon-specific variation in the signal of positive selection between gene lineages, both at MHC-IIA and MHC-IIB, and strikingly these differences were most apparent at the putative peptide-binding sites or in their immediate surroundings. This suggests that both gene lineages may have been subject to different regimes of pathogen-driven selection, possibly targeting different spectra of pathogenic antigens. Although we acknowledge that the hypothesis on the specialization of gene lineages in terms of antigen recognition is highly speculative, we recommend that it should be put to further investigation at the intra-specific level. So far, we are aware of intra-specific comparisons of selection at both gene lineages only in a single bird species, clearly showing sequence divergence and codon-specific differences in selection signal between the peptide-binding domains of the two gene lineages in the Leach’s storm petrel (57, 58). Interestingly, MHC-IIB sequences showed much greater sequence divergence between gene lineages than MHC-IIA and this divergence was found to be primarily driven by positive selection concentrating at the putative peptide-binding sites, suggesting possible functional differences in antigen recognition (58).

In conclusion, the results of this study provide novel insights into the macroevolution of MHC class II gene family in birds. By taking advantage of the rapidly accumulating high-quality genomic data, we showed that orthology of avian MHC-IIA genes has generally been retained throughout avian phylogeny (similarly to MHC-IIB), while the corresponding phylogenetic distribution of MHC-IIA and IIB gene lineages suggest their tight coevolution and advocates that the specific pairing of α and β chains in MHC-II heterodimers may have an adaptive significance. While our results advance knowledge on the avian MHC-II macroevolution, they also open exciting novel directions for future research on the functional role of the ancient MHC-II gene divergence and variation in evolutionary trajectories (e.g. duplication rate) between the two MHC-II gene lineages.

## Data availability statement

The original contributions presented in the study are included in the article/**Supplementary Material**. Further inquiries can be directed to the corresponding author.

## Author contributions

PM and WB designed the study. PM curated data and performed analyses;. PM and WB wrote the manuscript. PM, SVE, and WB revised the manuscript for intellectual content and approved the final version. All authors contributed to the article and approved the submitted version.

## Supplementary material

Appendix 1 GenBank numbers for MHC-IIA and MHC-IIB sequences used in this study.

Appendix 2 MHC-IIA and MHC-IIB sequences used in this study.

Appendix 3 Consensus Bayesian topologies for the upstream region (90 nt) of MHC class IIA and IIB exon 3 in Palaeognathae and 17 orders of non-passerine Neognathae birds.

Appendix 4 Consensus Bayesian topologies for the central and downstream region (186 nt) of MHC-IIA exon 3 in three selected orders of non-passerine birds showing the presence of both *DAA1* and *DAA2* gene lineages.
