# Supplementary material for: Ancient duplication, coevolution, and selection at the MHC class IIA and IIB genes of birds
Source: Front Immunol. 2023 Oct 27;14:1250824. doi: 10.3389/fimmu.2023.1250824 (PMC10641522; doi:10.3389/fimmu.2023.1250824)

Appendix 3

Ancient duplication, coevolution, and selection at the MHC class IIA and IIB genes of birds

Piotr Minias[1]*, Scott V. Edwards[2,3] and Wiesław Babik[4]

[1] University of Lodz, Faculty of Biology and Environmental Protection, Department of Biodiversity Studies and Bioeducation, Banacha 1/3, 90-237 Lodz, Poland

[2] Harvard University, Museum of Comparative Zoology, Cambridge, MA 02138, USA

[3] Harvard University, Department of Organismic and Evolutionary Biology, Cambridge, MA 02138, USA

[4] Jagiellonian University, Institute of Environmental Sciences, Faculty of Biology, Kraków, Poland

***Correspondence:**

Piotr Minias

pminias@op.pl

FIGURE S1 | Consensus Bayesian topologies of MHC class IIA and IIB sequences in Palaeognathae and 17 orders of non-passerine Neognathae birds. *DAA1* and *DAB1* clusters are marked in green, while *DAA2* and *DAB2* and marked in blue. Phylogenetic relationships were assessed for the upstream 90 nt region of MHC-II exon 3 (respective 30 aa region in Psittaciformes). Bayesian posterior probabilities were provided for major clusters. Reference sequences from *Bubo blakistoni* (GenBank nos. BJCB01040766 and BJCB01033660 for *DAA1* and *DAA2*, respectively; genome assembly GenBank no. GCA_004320225) and *Strix aluco* (GenBank nos. KJ162540 and KJ162542 for *DAB1* and *DAB2*, respectively) are bolded. *Andrias davidianus* was used as outgroup (Genbank no. KF611869 and KF723002 for MHC-IIA and IIB, respectively).

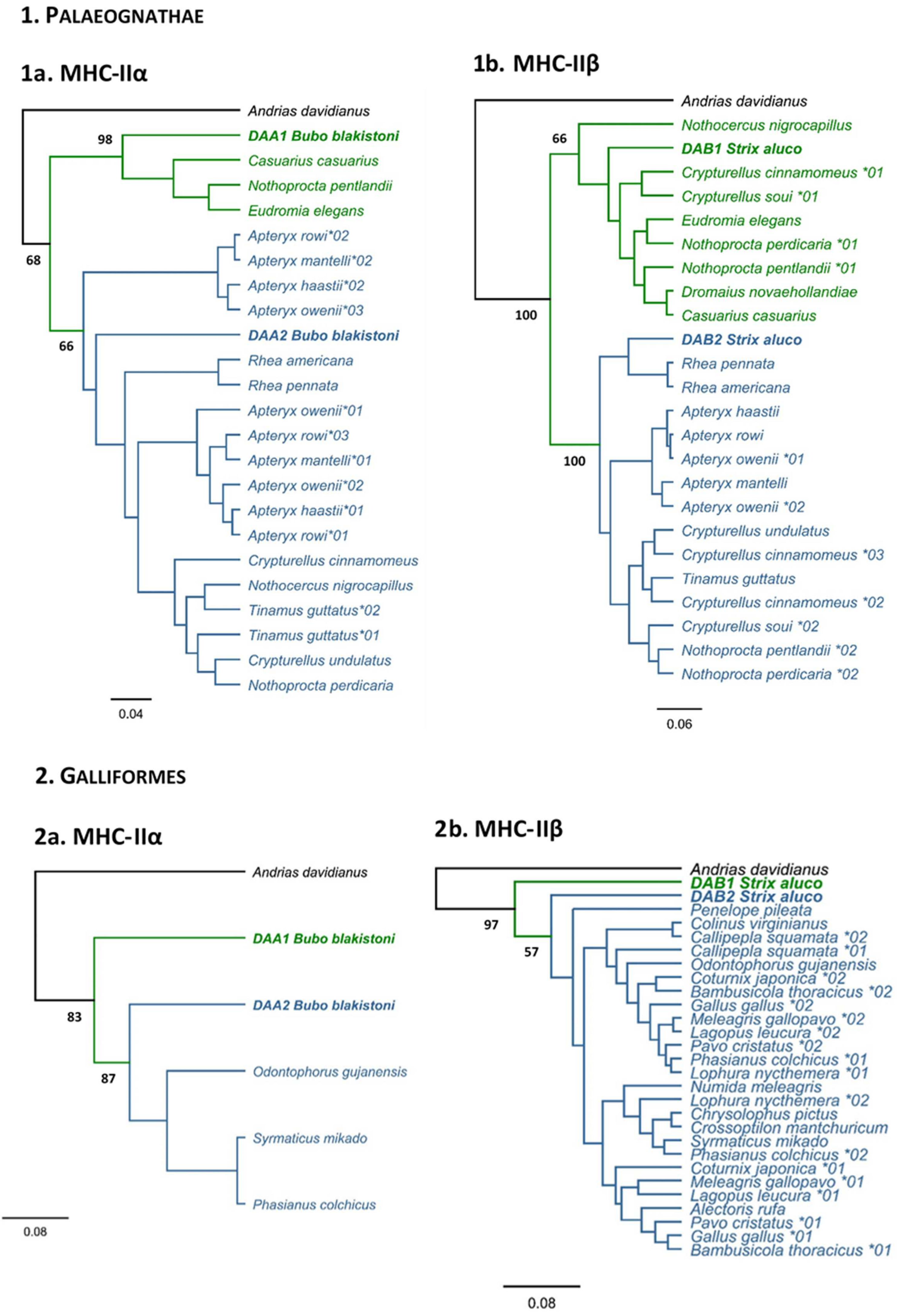

1. PALAEOGNATHAE
1a. MHC-IIα
Andrias davidianus
DAA1 Bubo blakistoni
Casuarius casuarius
Nothoprocta pentlandii
Eudromia elegans
Apteryx rowi*02
Apteryx mantelli*02
Apteryx haastii*02
Apteryx owenii*03
DAA2 Bubo blakistoni
Rhea americana
Rhea pennata
Apteryx owenii*01
Apteryx rowi*03
Apteryx mantelli*01
Apteryx owenii*02
Apteryx haastii*01
Apteryx rowi*01
Crypturellus cinnamomeus
Nothocercus nigrocapillus
Tinamus guttatus*02
Tinamus guttatus*01
Crypturellus undulatus
Nothoprocta perdicaria
98
68
66
0.04
1b. MHC-IIβ
Andrias davidianus
Nothocercus nigrocapillus
DAB1 Strix aluco
Crypturellus cinnamomeus *01
Crypturellus soui *01
Eudromia elegans
Nothoprocta perdicaria *01
Nothoprocta pentlandii *01
Dromaius novaehollandiae
Casuarius casuarius
DAB2 Strix aluco
Rhea pennata
Rhea americana
Apteryx haastii
Apteryx rowi
Apteryx owenii *01
Apteryx mantelli
Apteryx owenii *02
Crypturellus undulatus
Crypturellus cinnamomeus *03
Tinamus guttatus
Crypturellus cinnamomeus *02
Crypturellus soui *02
Nothoprocta pentlandii *02
Nothoprocta perdicaria *02
66
100
100
0.06
2. GALLIFORMES
2a. MHC-IIα
Andrias davidianus
DAA1 Bubo blakistoni
DAA2 Bubo blakistoni
Odontophorus gujanensis
Syrmaticus mikado
Phasianus colchicus
83
87
0.08
2b. MHC-IIβ
Andrias davidianus
DAB1 Strix aluco
DAB2 Strix aluco
Penelope pileata
Colinus virginianus
Callipepla squamata *02
Callipepla squamata *01
Odontophorus gujanensis
Coturnix japonica *02
Bambusicola thoracicus *02
Gallus gallus *02
Meleagris gallopavo *02
Lagopus leucura *02
Pavo cristatus *02
Phasianus colchicus *01
Lophura nycthemera *01
Numida meleagris
Lophura nycthemera *02
Chrysolophus pictus
Crossoptilon mantchuricum
Syrmaticus mikado
Phasianus colchicus *02
Coturnix japonica *01
Meleagris gallopavo *01
Lagopus leucura *01
Alectoris rufa
Pavo cristatus *01
Gallus gallus *01
Bambusicola thoracicus *01
97
57
0.08

3. Anseriformes

3a. MHC-IIα

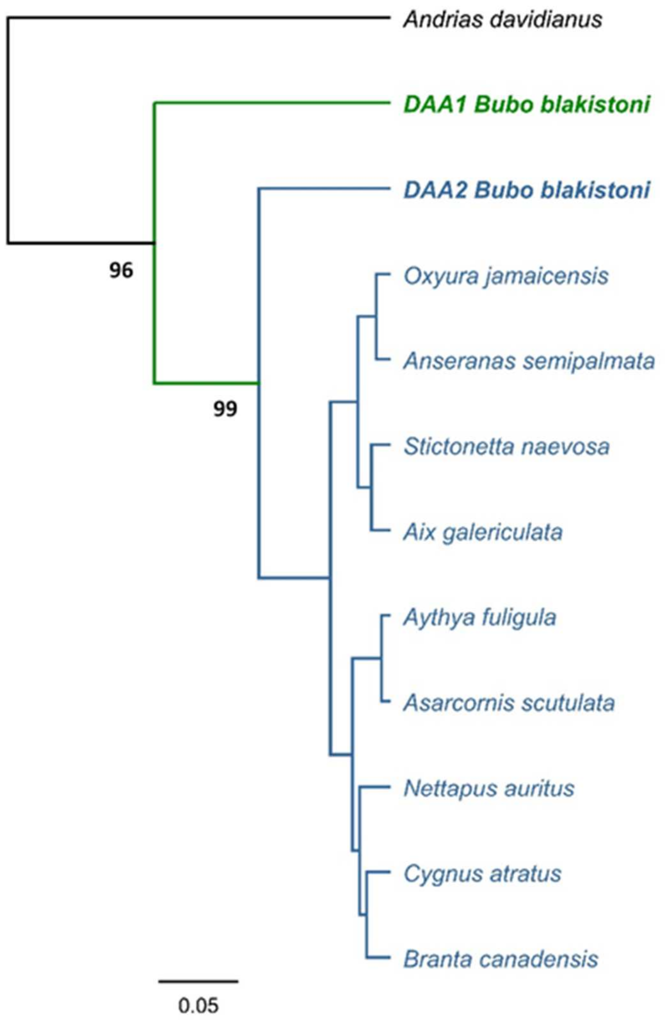

3b. MHC-IIβ

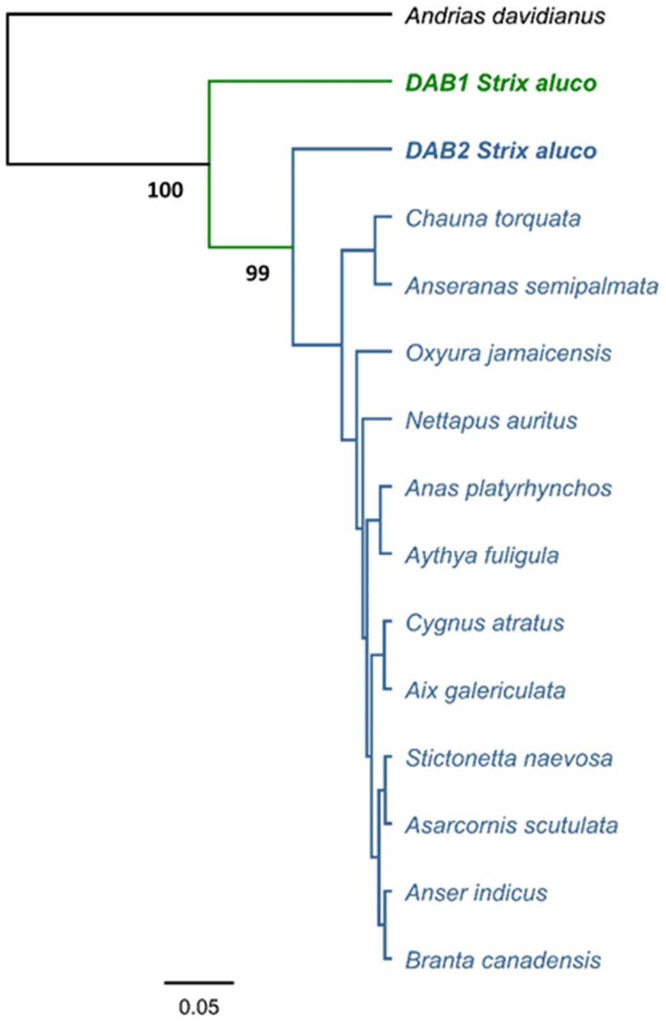

4. Pterocliformes

4a. MHC-IIα

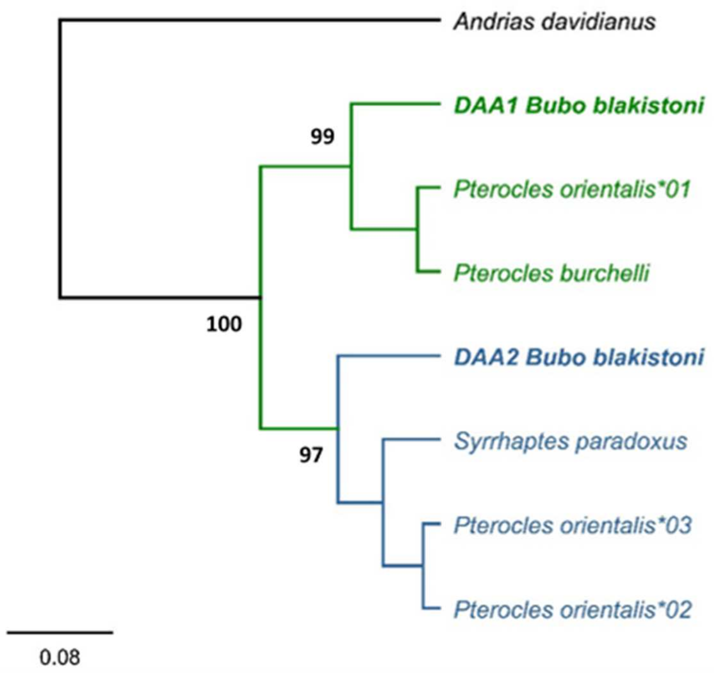

4b. MHC-IIβ

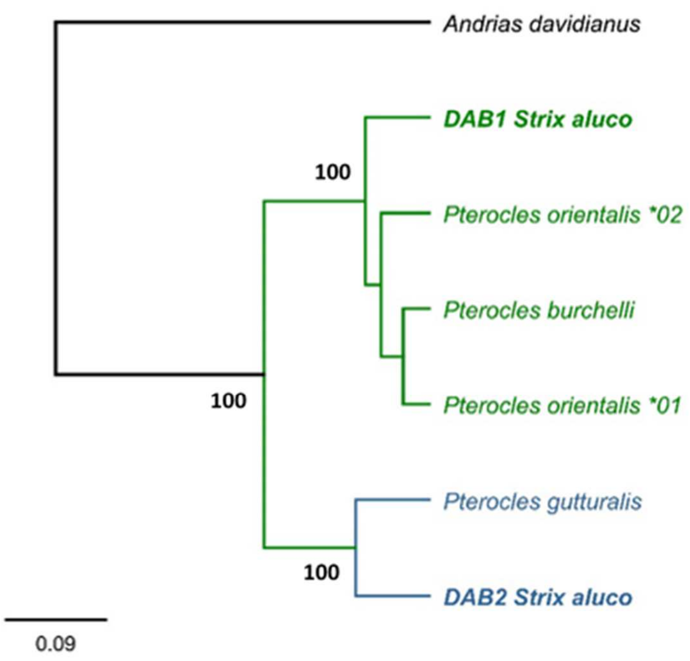

5. Otidiformes

5a. MHC-IIα

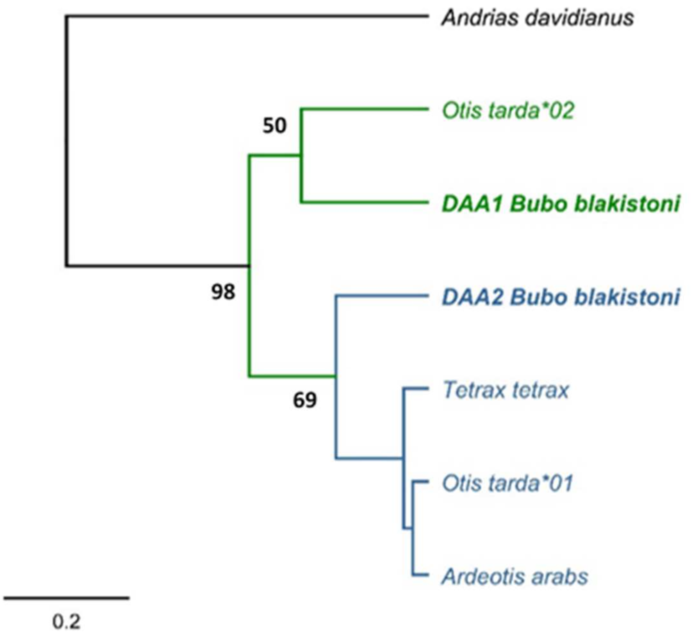

5b. MHC-IIβ

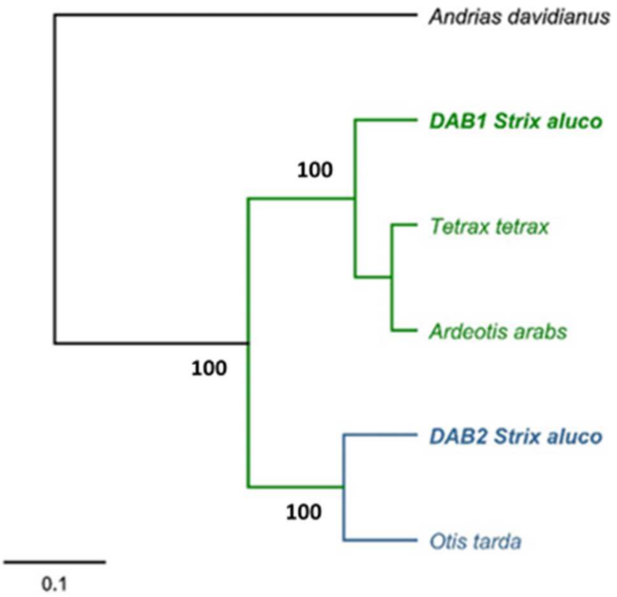

6. Caprimulgiformes

6a. MHC-IIα

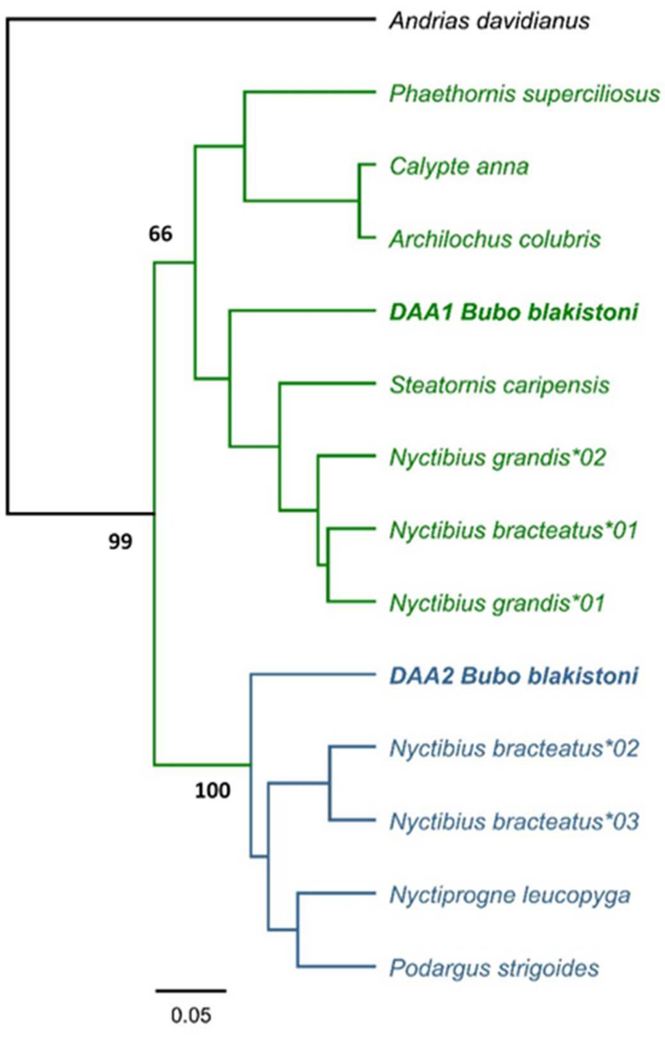

6b. MHC-IIβ

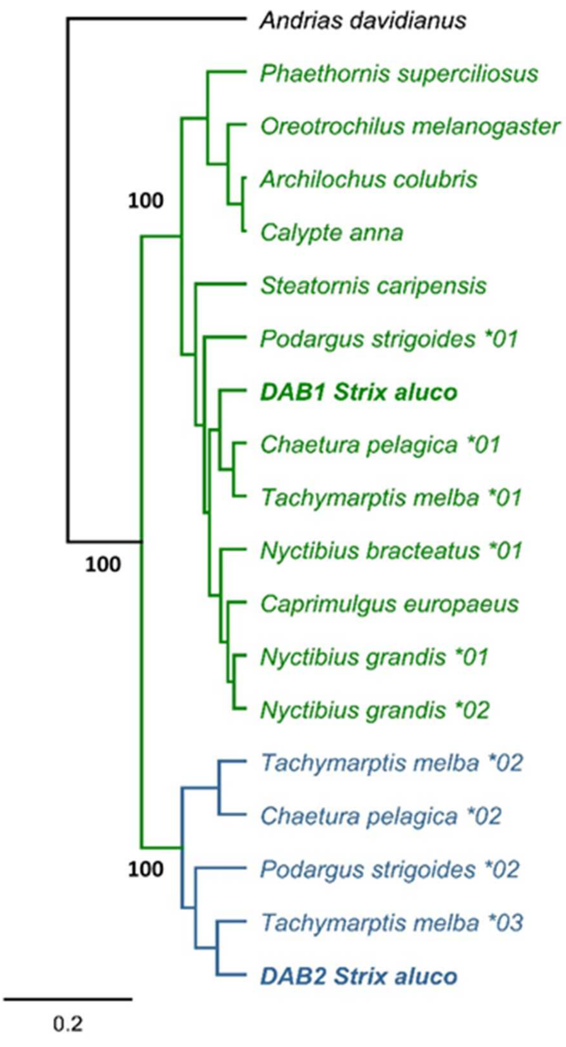

7. Gruiformes

7a. MHC-IIα

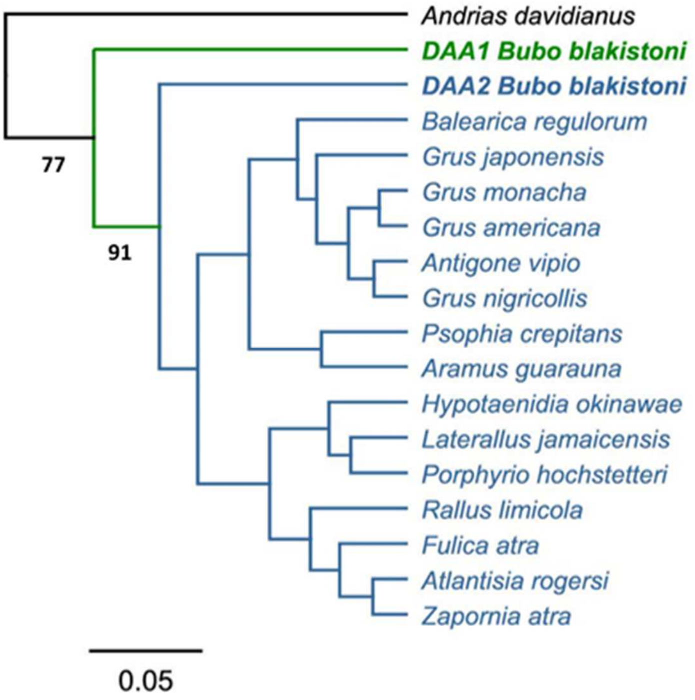

7b. MHC-IIβ

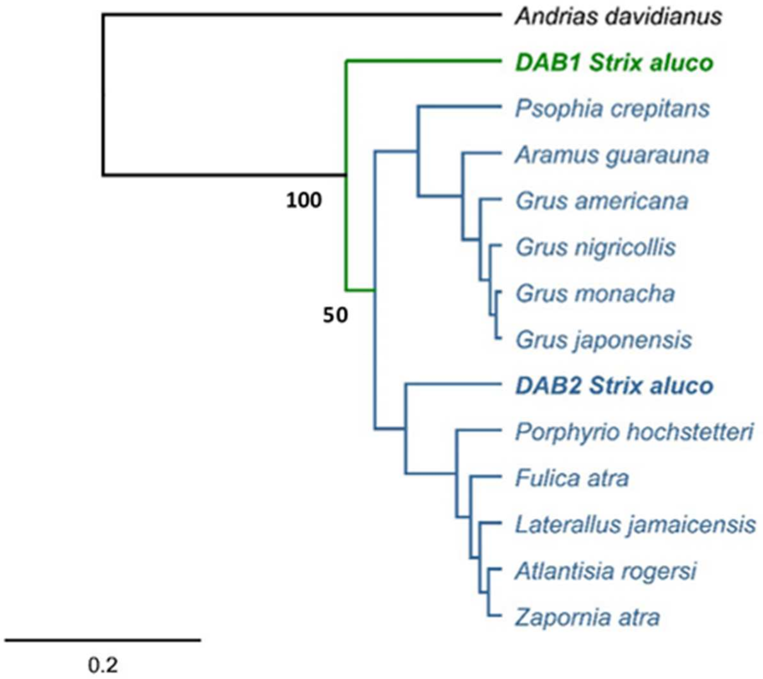

8. Charadriiformes

8a. MHC-IIα

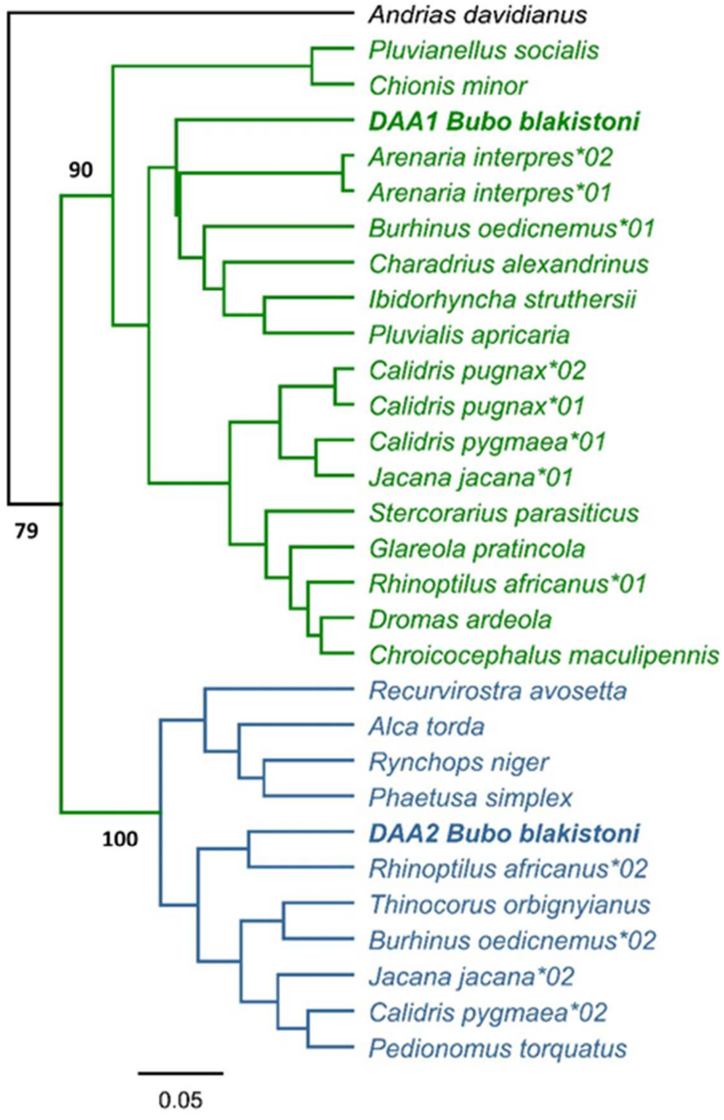

8b. MHC-IIβ

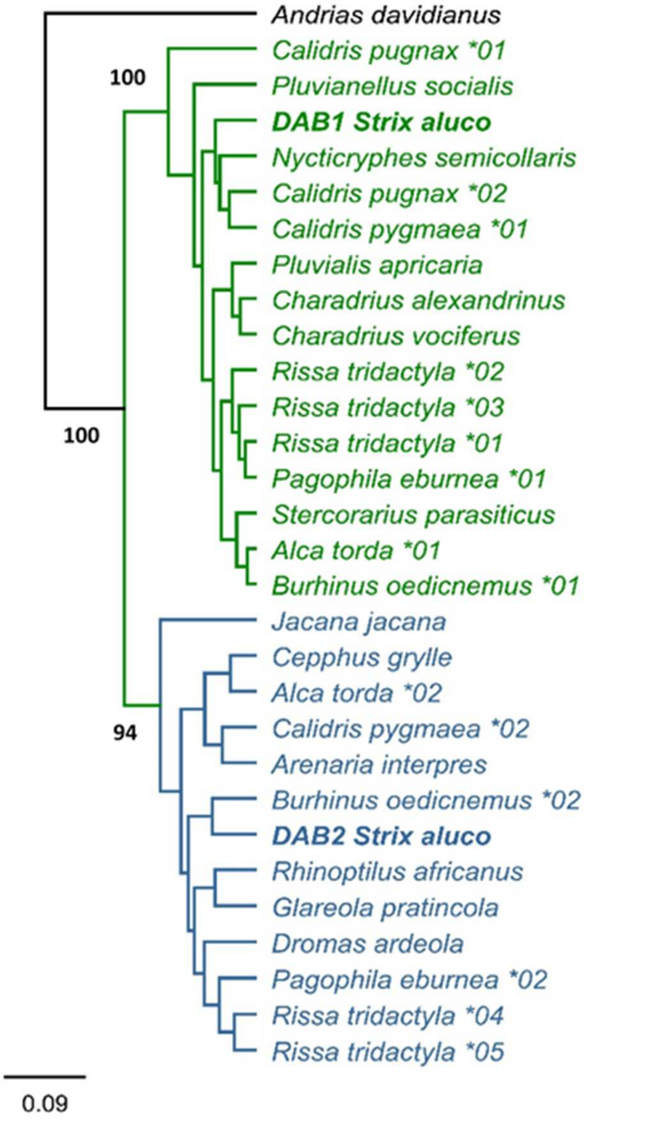

9. Sphenisciformes

9a. MHC-IIα

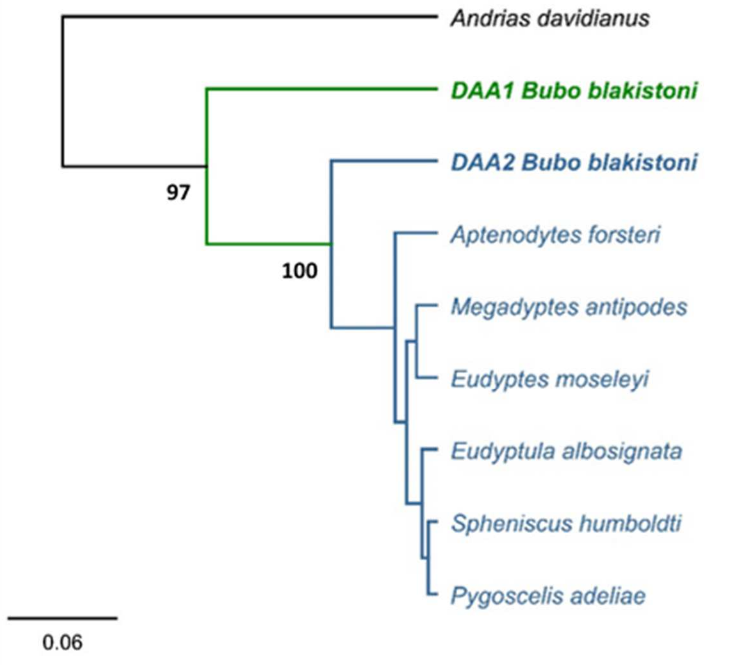

9b. MHC-IIβ

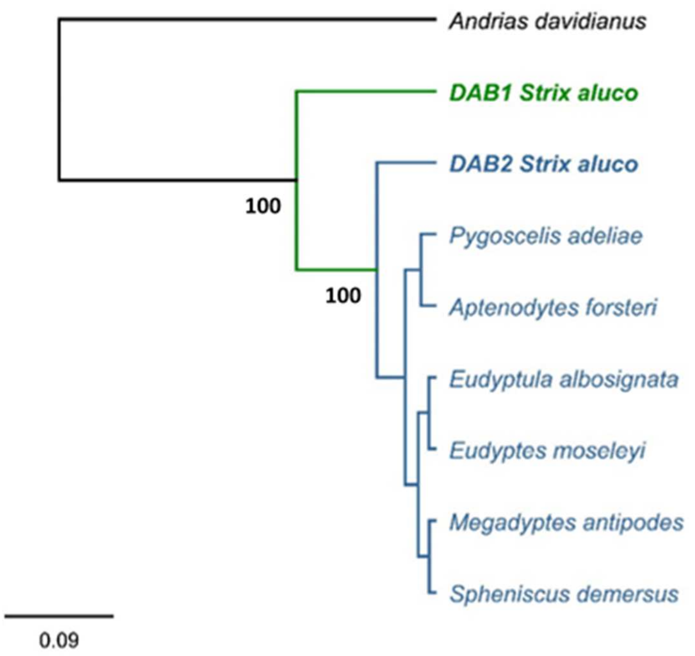

10. Procellariiformes

10a. MHC-IIα

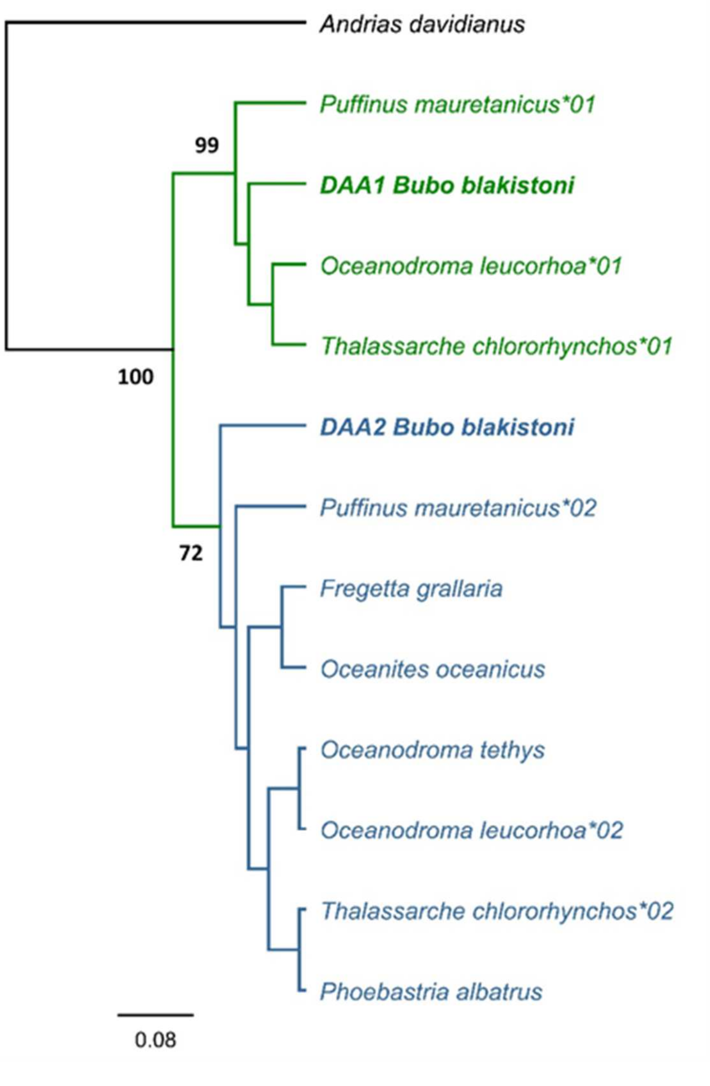

10b. MHC-IIβ

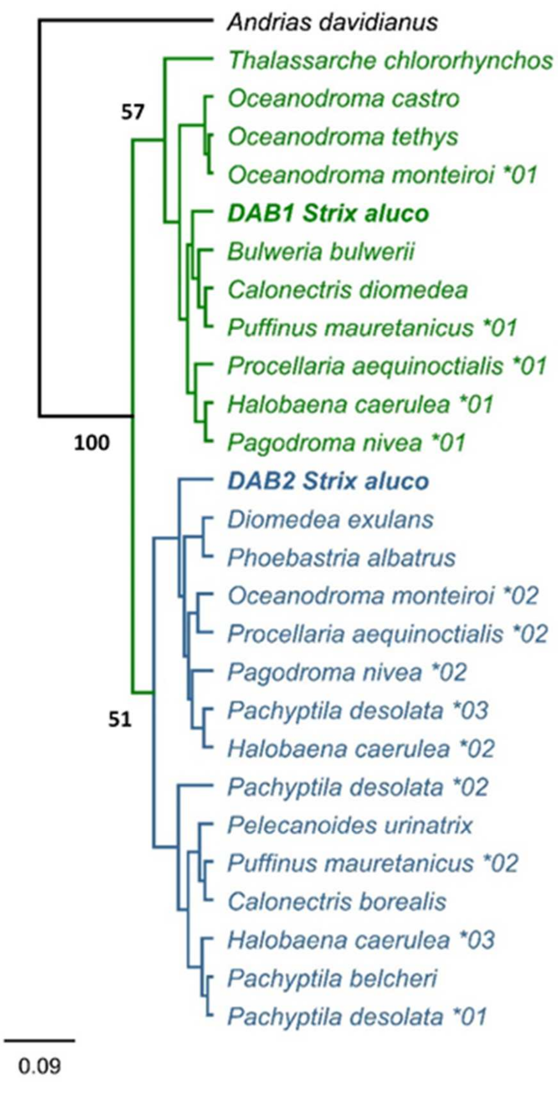

11. Suliformes

11a. MHC-IIα

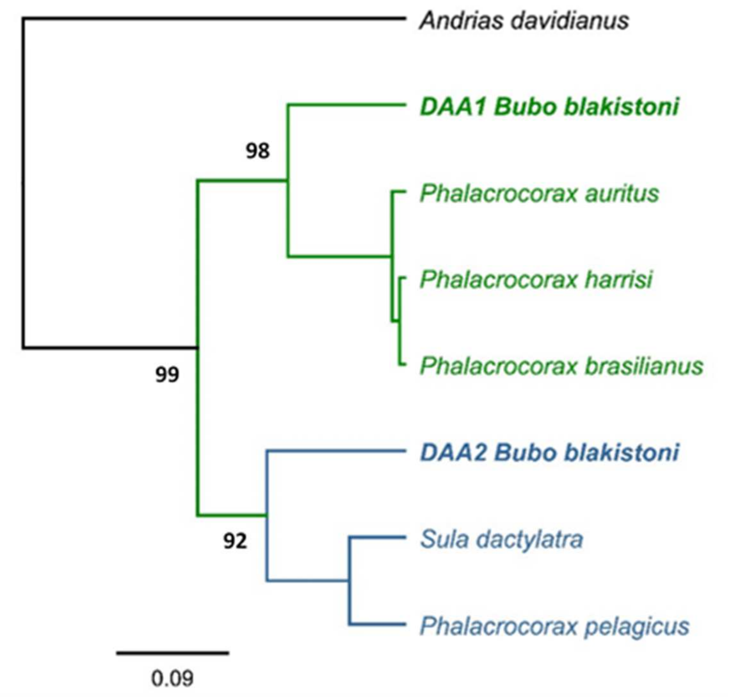

11b. MHC-IIβ

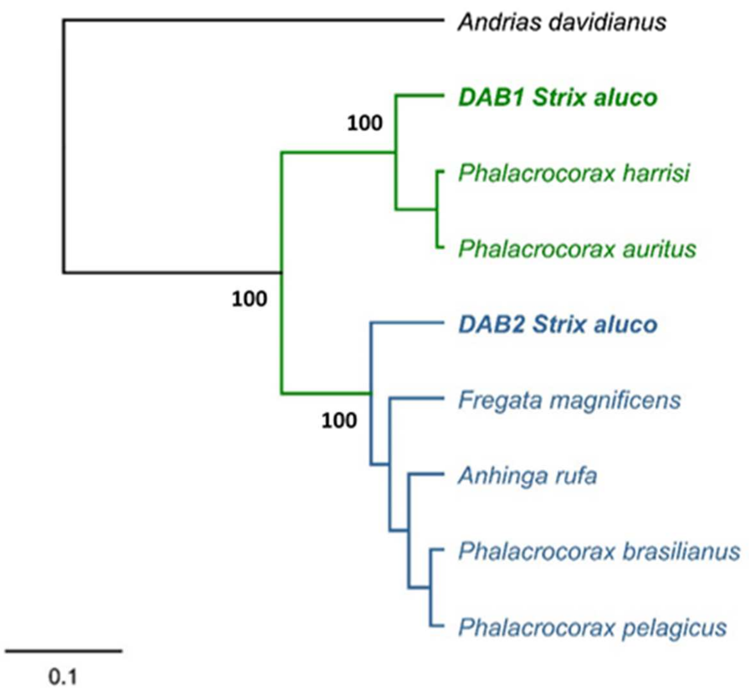

12. Pelecaniformes

12a. MHC-IIα

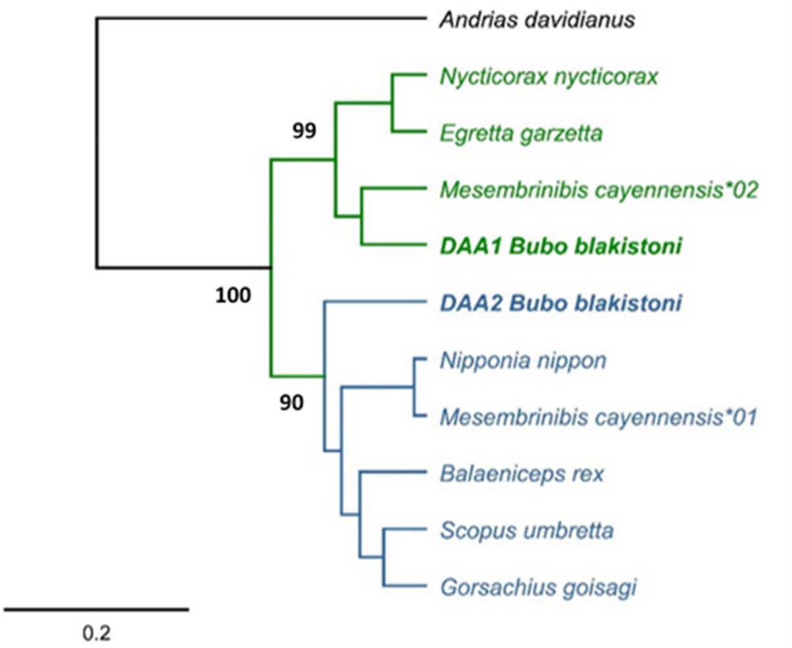

12b. MHC-IIβ

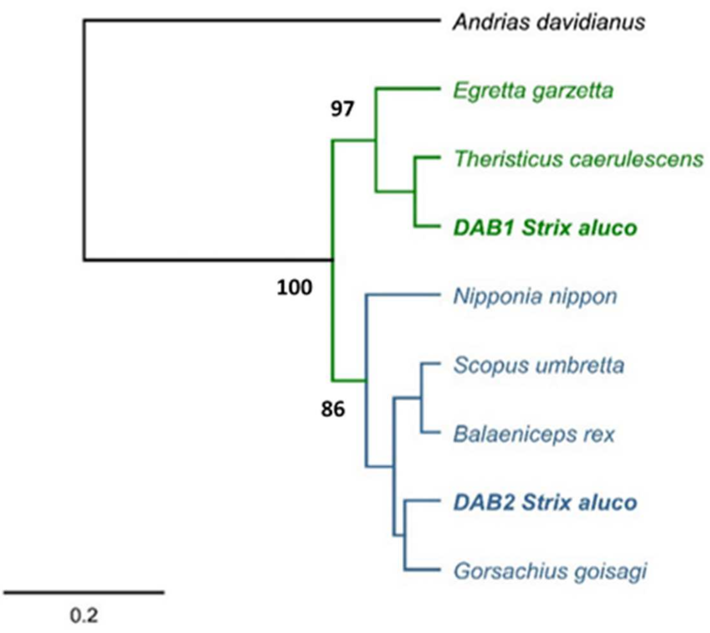

13. Accipitriformes

13a. MHC-IIα

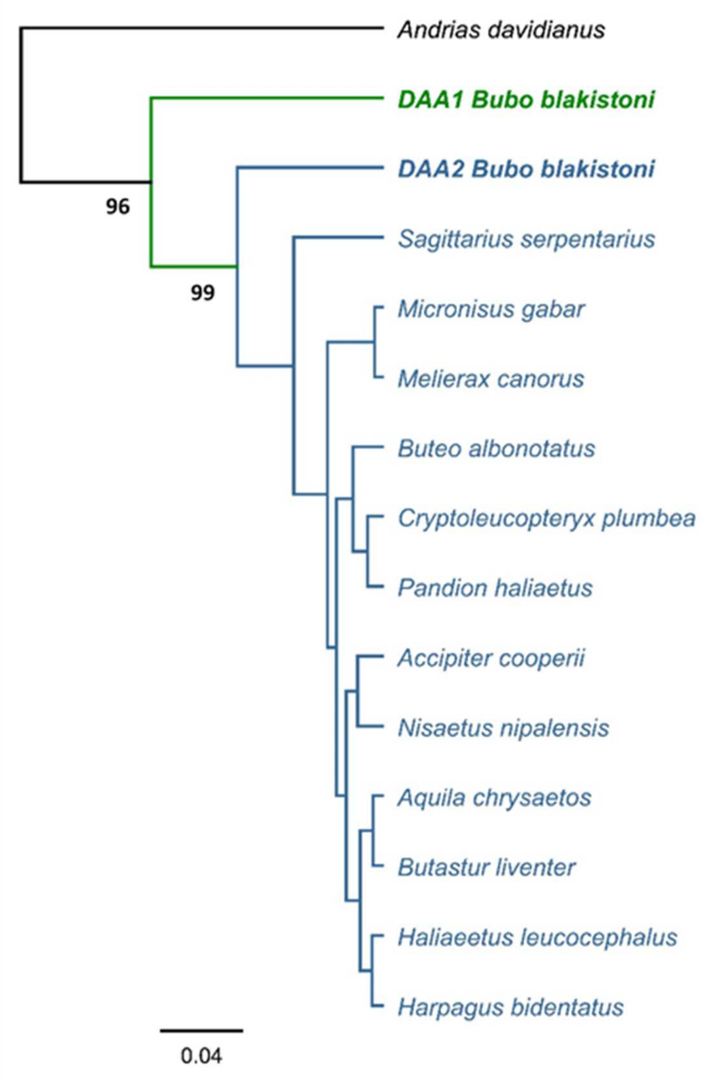

13b. MHC-IIβ

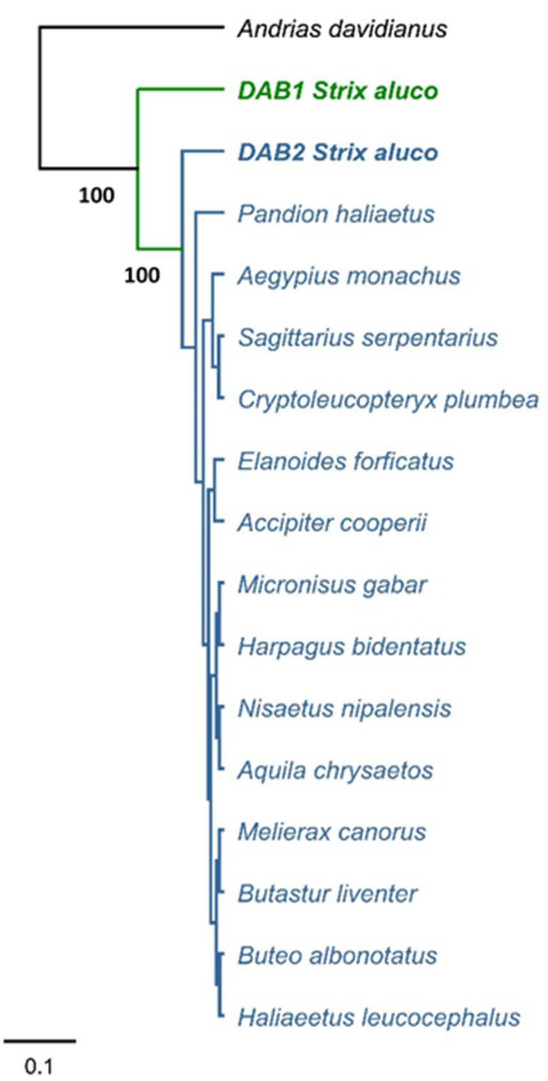

14. Strigiformes

14a. MHC-IIα

Shown in Fig. 1

14b. MHC-IIβ

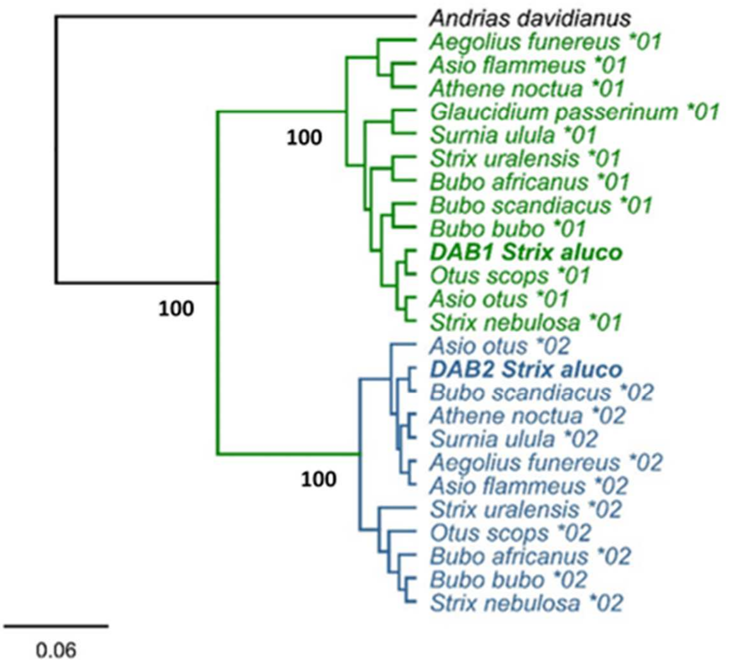

15. Piciformes

15a. MHC-IIα

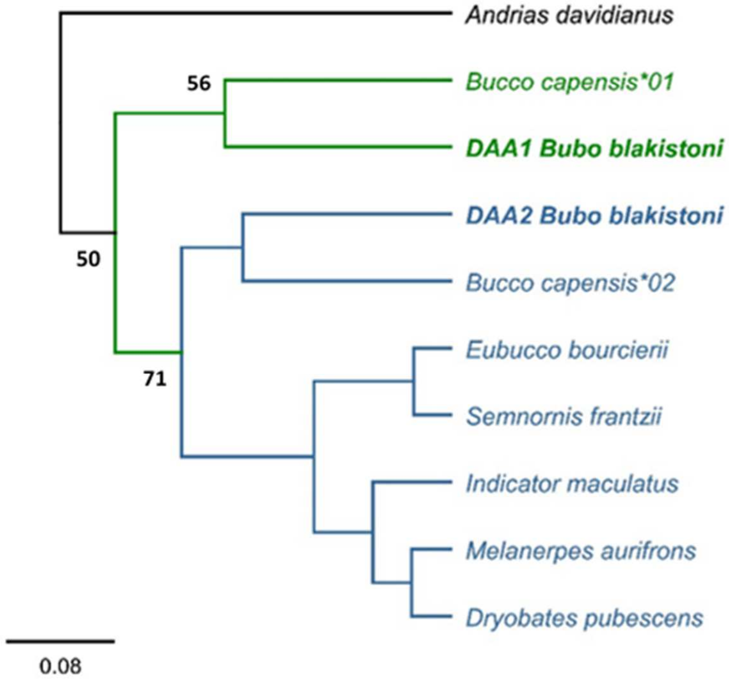

15b. MHC-IIβ

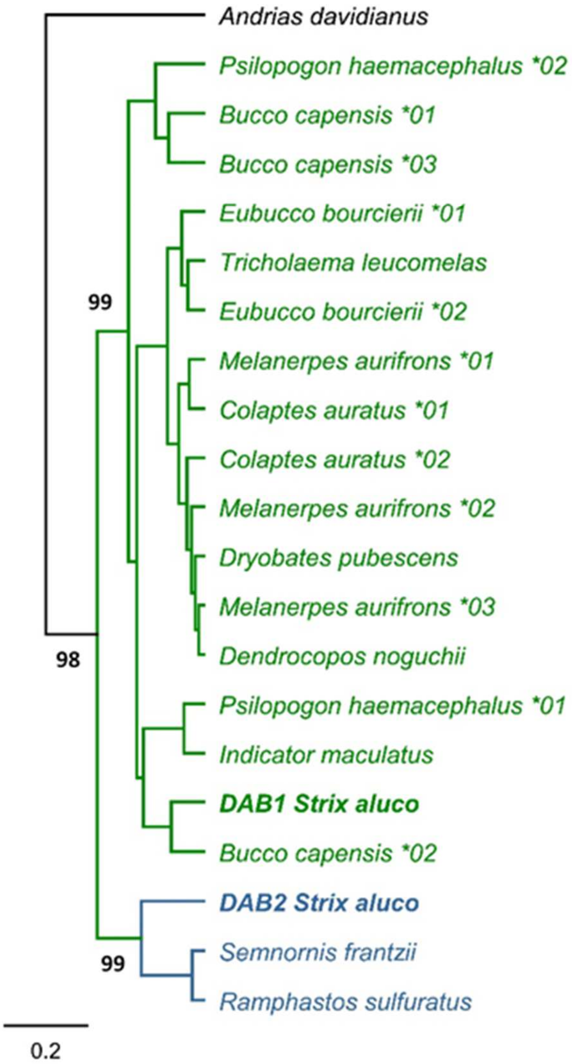

16. Coraciiformes

16a. MHC-IIα

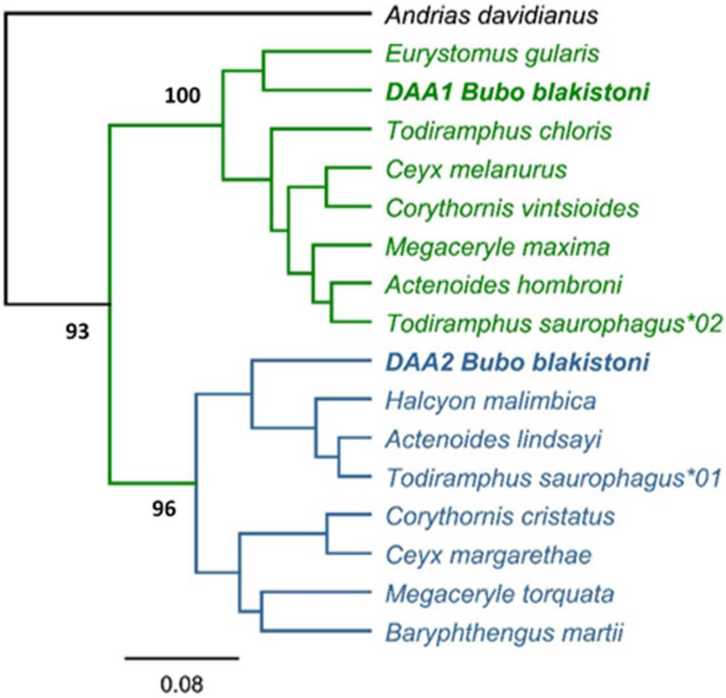

16b. MHC-IIβ

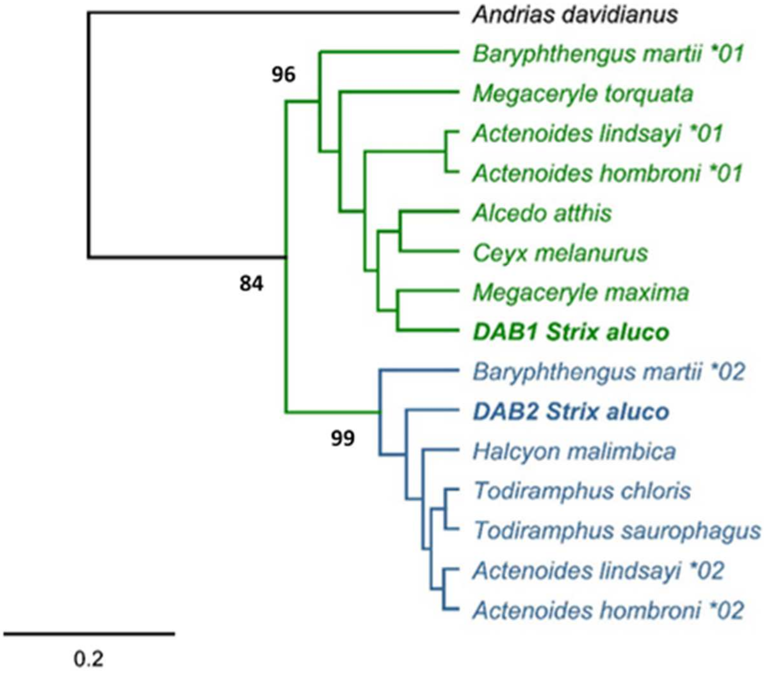

17. Falconiformes

17a. MHC-IIα

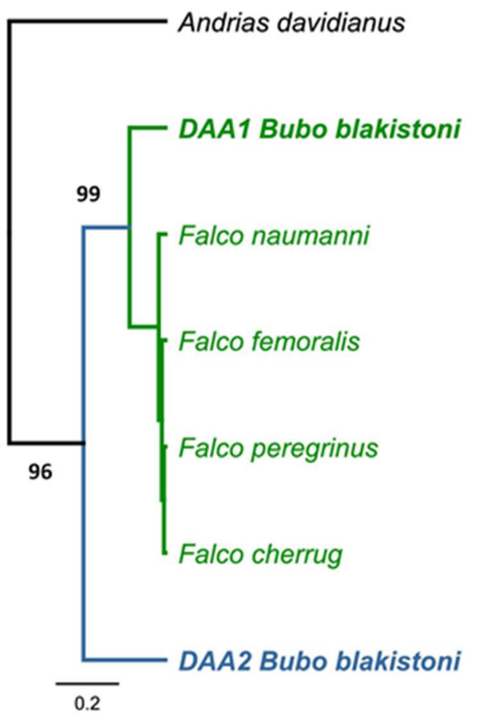

17a. MHC-IIβ

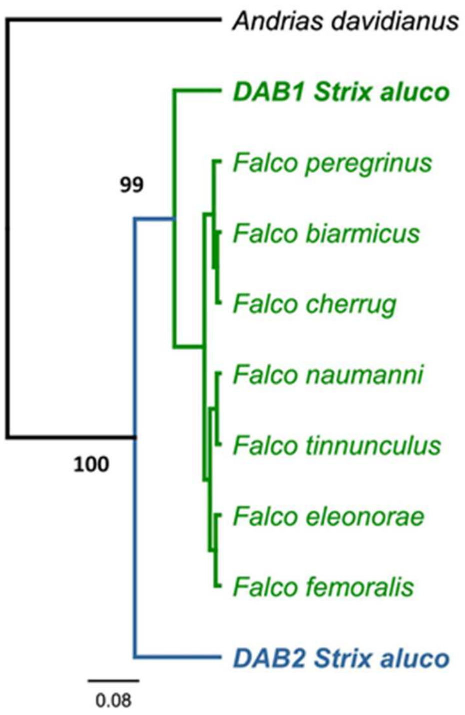

18. Psittaciformes

18a. MHC-IIα

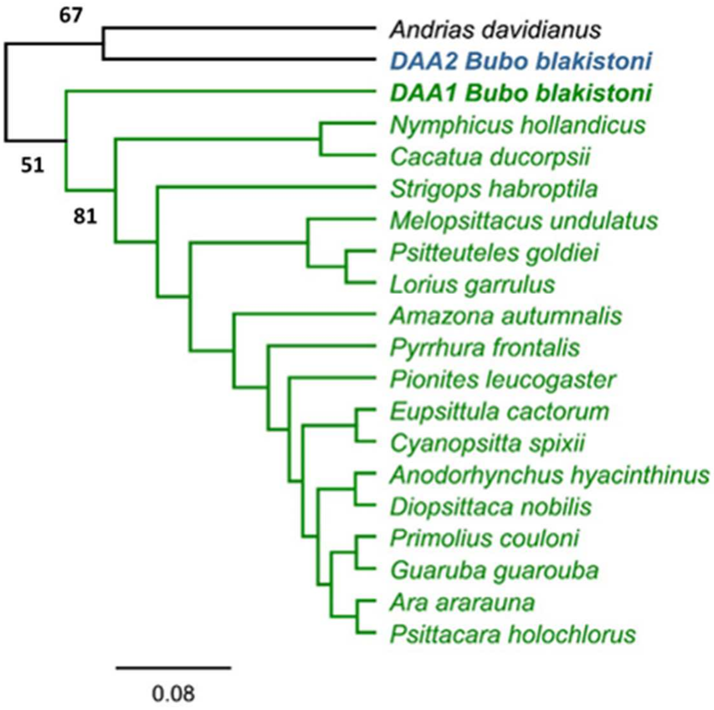

18b. MHC-IIβ

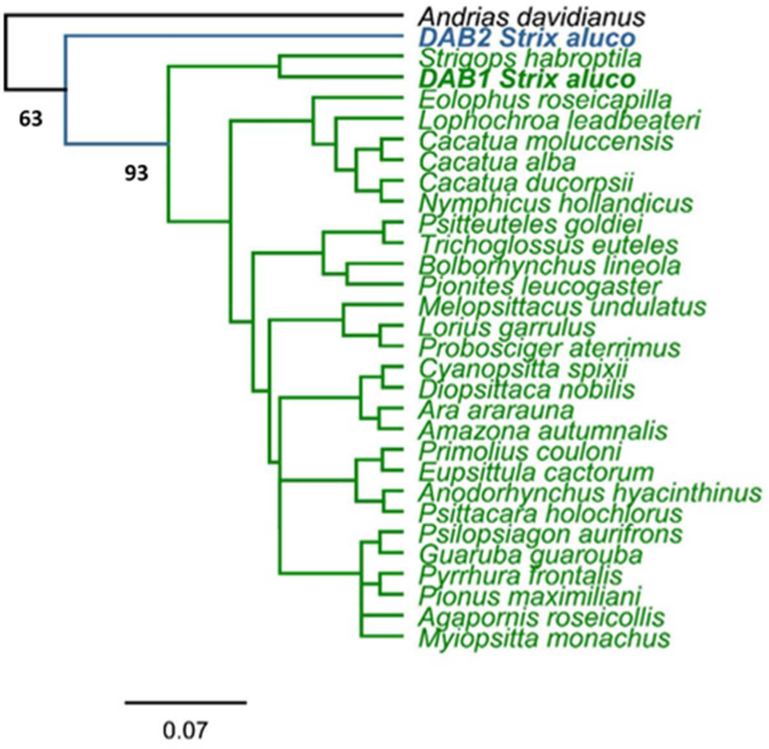

Supplement: Appendix 1 — GenBank numbers for MHC-IIA and MHC-IIB sequences used in this study. [file DataSheet_1.pdf]
