# Supplementary material for: Ancient duplication, coevolution, and selection at the MHC class IIA and IIB genes of birds
Source: Front Immunol. 2023 Oct 27;14:1250824. doi: 10.3389/fimmu.2023.1250824 (PMC10641522; doi:10.3389/fimmu.2023.1250824)

Appendix 4

Ancient duplication, coevolution, and selection at the MHC class IIA and IIB genes of birds

Piotr Minias[1]*, Scott V. Edwards[2,3] and Wiesław Babik[4]

[1] University of Lodz, Faculty of Biology and Environmental Protection, Department of Biodiversity Studies and Bioeducation, Banacha 1/3, 90-237 Lodz, Poland

[2] Harvard University, Museum of Comparative Zoology, Cambridge, MA 02138, USA

[3] Harvard University, Department of Organismic and Evolutionary Biology, Cambridge, MA 02138, USA

[4] Jagiellonian University, Institute of Environmental Sciences, Faculty of Biology, Kraków, Poland

***Correspondence:**

Piotr Minias

pminias@op.pl

FIGURE S2 | Consensus Bayesian topologies of MHC class IIA sequences in three selected orders of non-passerine birds showing the presence of both *DAA1* and *DAA2* gene lineages. *DAA1* and *DAA2* sequences are marked in green and blue, respectively. Phylogenetic relationships were assessed for the central and downstream region (186 nt) of MHC-IIA exon 3. Bayesian posterior probabilities (>0.50) were provided for major clusters. Assignment of sequences to either *DAA1* or *DAA2* gene lineage was based on the phylogenetic analysis of the upstream region (90 nt) of MHC-IIA exon 3 (see Appendix 1). *Andrias davidianus* was used as outgroup (Genbank no. KF611869).

1. CHARADRIIFORMES

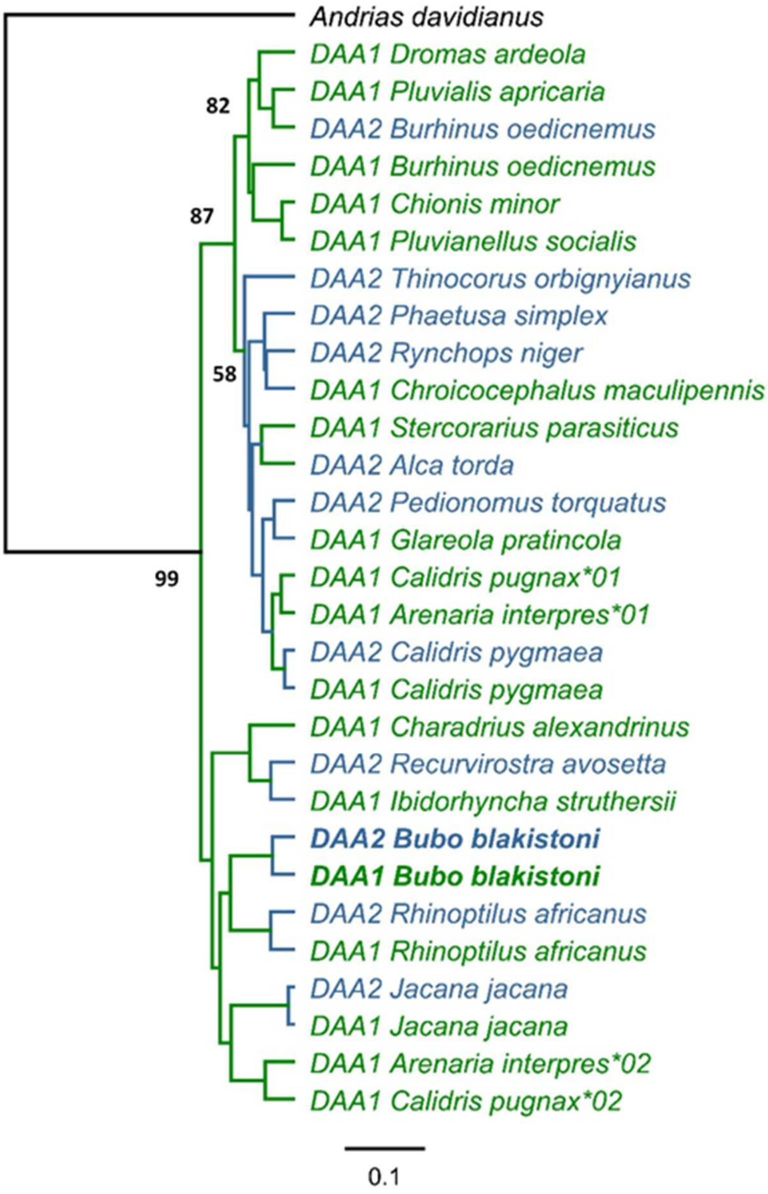

2. PROCELLARIIFORMES

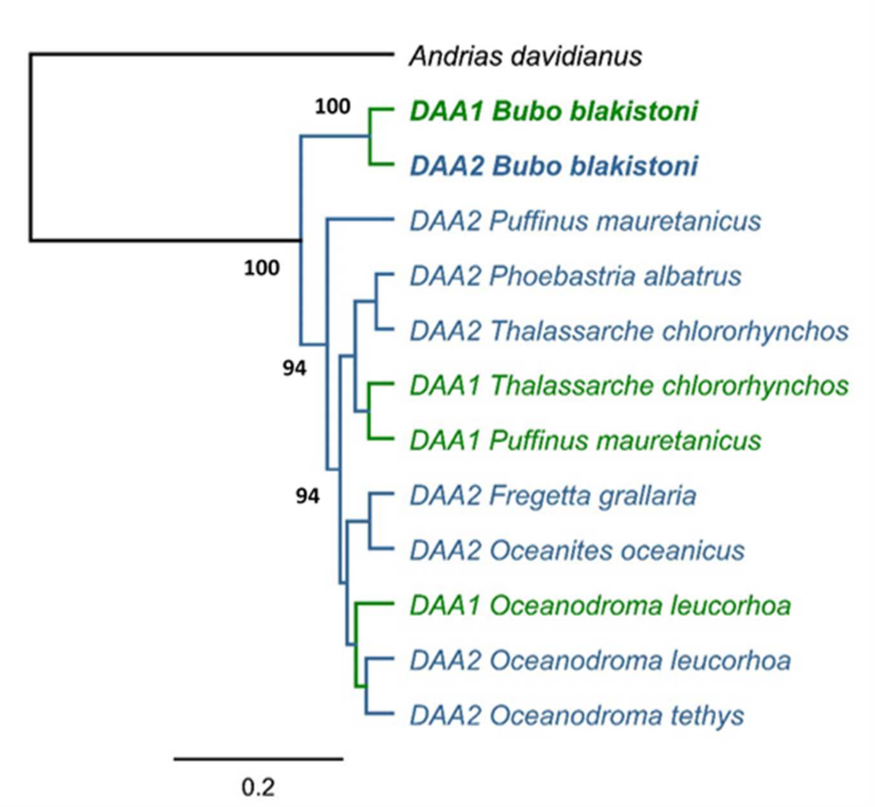

3. CORACIIFORMES

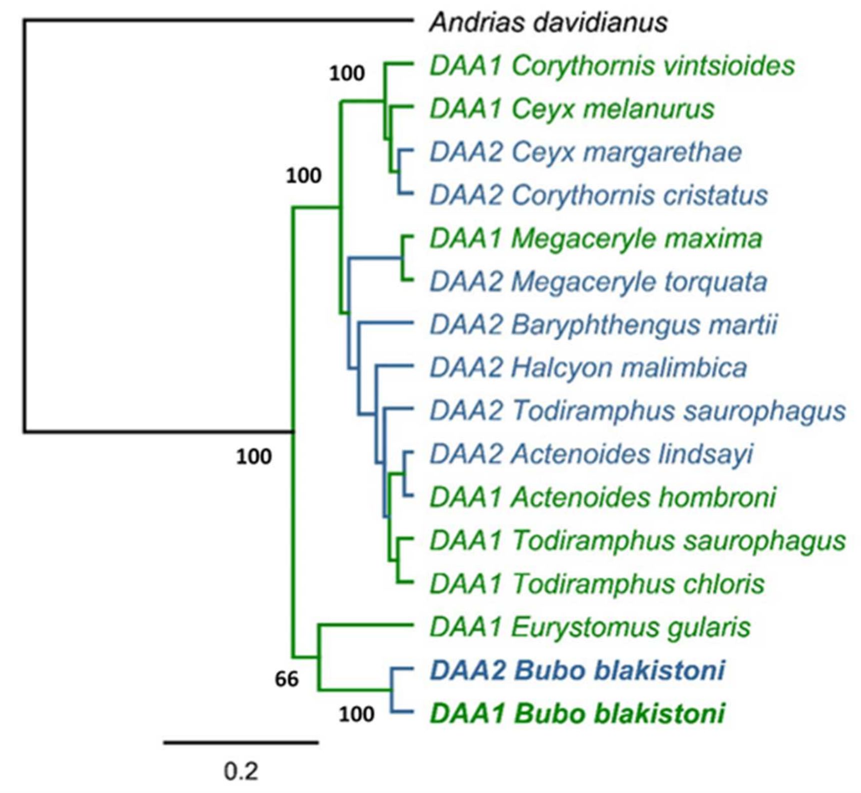

Supplement: Appendix 2 — MHC-IIA and MHC-IIB sequences used in this study. [file DataSheet_2.pdf]
